# Clinically meaningful interpretability of an AI model for ECG classification

**DOI:** 10.1038/s41746-025-01467-8

**Published:** 2025-02-17

**Authors:** Vadim Gliner, Idan Levy, Kenta Tsutsui, Moshe Rav Acha, Jorge Schliamser, Assaf Schuster, Yael Yaniv

**Affiliations:** 1 Computer Science Department, Technion-IIT, Haifa, Israel; 2 Saitama Medical University International Medical Center, Saitama, Japan; 3 Cardiology Department, Shaare Zedek Medical Center, Jerusalem, Israel; 4 Cardiology Department, Lady David Carmel Medical Center, Haifa, Israel; 5 Laboratory of Bioenergetic and Bioelectric Systems, Biomedical Engineering Faculty, Technion-IIT, Haifa, Israel

**Keywords:** Cardiovascular diseases, Arrhythmias

## Abstract

Despite the high accuracy of AI-based automated analysis of 12-lead ECG images for classification of cardiac conditions, clinical integration of such tools is hindered by limited interpretability of model recommendations. We aim to demonstrate the feasibility of a generic, clinical resource interpretability tool for AI models analyzing digitized 12-lead ECG images. To this end, we utilized the sensitivity of the Jacobian matrix to compute the gradient of the classifier for each pixel and provide medical relevance interpretability. Our methodology was validated using a dataset consisting of 79,226 labeled scanned ECG images, 11,316 unlabeled and 1807 labeled images obtained via mobile camera in clinical settings. The tool provided interpretability for both morphological and arrhythmogenic conditions, highlighting features in terms understandable to physician. It also emphasized significant signal features indicating the absence of certain cardiac conditions. High correlation was achieved between our method of interpretability and gold standard interpretations of 3 electrophysiologists.

## Introduction

The 12-lead electrocardiography (ECG) serves as the primary clinical tool for detecting both morphological and arrhythmogenic cardiac conditions. In the USA, out of 131 million visits to emergency rooms in 2020, over 32 million ECG checks were performed, and out of one billion visits to community health centers, 47 million ECG checks were performed. Algorithms for the detection of cardiac conditions from 12-lead ECG images have been developed, including by our team^1^ and others^2–5^. However, despite the high accuracy achieved by AI-based automated classifications of cardiac conditions using 12-lead ECG images, their integration into clinical practice is impeded by the limited justification of the model output provided to the physician^6^. This situation hinders the adoption of such tools as an intuitive understanding of the model results is needed to develop trust in its recommendations. Introducing interpretability to the 12-lead ECG classifier is thus crucial for garnering trust and acceptance of clinical teams, while also facilitating the detection of errors in the resulting diagnostics.

To enhance intuitiveness, the interpretability of the model classifications of cardiac conditions should be provided to physicians in their own “language”, namely, in the form of ECG features in the input data that have the most significant influence on the resulting diagnosis^6^. Furthermore, ideally, these features should correlate with the well-established characteristics of the cardiac conditions as documented in the existing literature^7^ and used by physicians in clinical practice. However, it is also important to mention that there is a lack of consensus with regards to the biomarkers of cardiac conditions^8^.

Efforts to provide interpretability to the 12-lead ECG classifier initially focused on artificial images generated from signals (each ECG lead is a time series of scalar measurements that we call “a signal”); see ref. ^9^ for a review of interpretability methods. One interpretability approach utilized linear interpretable model-agnostic explanations (LIME)^10^. However, LIME and other methods^11,12^ operate directly on the signal rather than on the image, necessitating signal extraction, which poses a significant challenge and introduces an additional source of inaccuracy^9^. For instance, they may highlight the entire QRS complex irrespective of the underlying heart condition, potentially resulting in inaccurate diagnoses.

Recent efforts have enhanced interpretability for the 12-lead ECG classifier by using high-quality scanned 12-lead ECG images as input. For example, GRAD-CAM generates heatmaps highlighting crucial regions influencing the model classification^12^ (Supplementary Fig. 1). Although GRAD-CAM has been employed in various studies (refer to the list in ref. ^9^), it has limitations that constrain its clinical practice. One primary drawback is that highlighted features do not consistently align with diagnostic criteria^9^. Additionally, GRAD-CAM may overlook clinical features that are as small as several pixels and fall below the classifier handling resolution but not of humans in manual interpretation. It also often lacks specificity, emphasizing the image background rather than the clinically meaningful ECG signal (see Supplementary Fig. 1). Moreover, GRAD-CAM relies on the final layer of the convolutional neural network, typically designed for high-level features like texture, shape and structure and not 12-lead ECG features.

The above interpretability efforts have been focusing on high-quality scanned 12-lead ECG images as input. Mobile cameras, such as those on smartphones, offer a practical means of digitizing 12-lead ECG machine paper printouts commonly found in clinics. They also enable copying of images from screens, which will facilitate sharing, for example, during consultations and remote healthcare. Furthermore, smartphones are among the few devices that are almost always accessible to physicians. However, photographed images often exhibit artifacts, tilting, skewing, crumpling and may include shadowing^13^. Therefore, interpretability methods that are effective on photographed images is a considerable advantage, but also pose a substantial challenge.

Here, we introduce a high-resolution ECG interpretability tool designed for photographed images. The proposed interpretability algorithm is based on the sensitivity of the Jacobian matrix. Our method offers several advantages over existing methods. Firstly, it demonstrates interpretability at the pixel level, achieving the highest possible resolution. The method is also sensitive to fine features and highlights signals within the image, rather than the background. The approach is format-agnostic and generic, requiring no additional modifications to the network. In addition, it is also capable of explaining why certain conditions do not exist.

## Results

### Overview

**Fig. 1:**
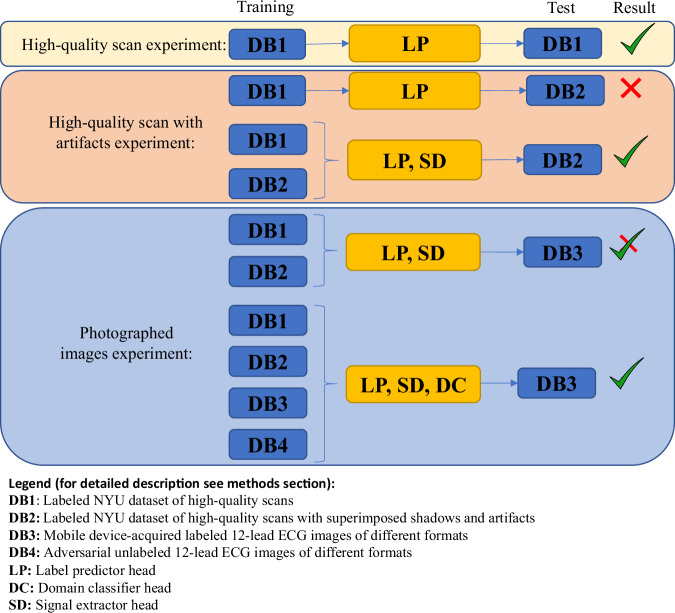
Schematic diagram of experiments performed in this work. The system was trained and tested on four different databases using different configurations. A green check mark indicates good system performance, while a red cross indicates low performance.

Our primary objective was to establish a clinically acceptable interpretation method that is applicable on general 12-lead ECG images, including those photographed using a mobile (smartphone) camera. To demonstrate the method’s clinical viability and independence from network architecture, the results section outlines three sets of experiments (refer to Fig. 1). We leveraged the sensitivity of the Jacobian matrix to calculate the gradient of the classifier for each pixel and provide interpretability within the medical domain. To achieve clinically acceptable interpretation method, we conducted tests on our previously published ECG-Adversarial network 1 (herein, the network), exclusively utilizing the label predictor and excluding the domain classifier model. The ECG-Adversarial network employs a domain adversarial training approach^14^ that derives domain-agnostic features that perform well on both source and target domains, a strategy that enhances model robustness and adaptability, particularly to images from clinical practice, as explained below.

In the first experiment, the performance of our approach was assessed on the labeled New York University (NYU) dataset (herein, DB1, available at https://education.med.nyu.edu/ecg-database/app), consisting of high-quality scan images. As expected, the interpretability excelled in the high-quality scan experiment. In the second experiment, the performance was evaluated on the labeled NYU dataset with shadows and artifacts (DB2), as detailed in the methods section. As expected, the network demonstrated suboptimal performance and consequently yielded inferior interpretability results when tested on images with shadows and artifacts because they were taken from a distribution different from that of its training set (high-quality scans with no shadows and artifacts).

Consider a neural network whose function is to classify a specific cardiac condition when given a 12-lead ECG image. The Jacobian matrix, being a set of partial derivatives of the network output, relies on the network accuracy. If the network output lacks precision, the partial derivatives will also be inaccurate. Therefore, the network’s accuracy is pivotal for interpretability.

To solve the problem on images with shadows and artifacts taken by mobile cameras, we expanded the network architecture to incorporate not only the label predictor but also a signal decoder. The signal decoder is a convolutional neural network consisting of several layers, including transposed convolutional layers, which perform the opposite operation of a regular convolution. It was purposefully designed to extract signals from the 12-lead ECG image. This addition aimed to bolster the network resilience in handling inputs with shadows and artifacts because it extracts relevant features from ECG images while minimizing information loss, especially in areas with complex patterns, such as shadows.

In the third experiment, we assessed the performance of the network with the modified architecture on a third dataset (DB3) consisting of images that were photographed using a smartphone in the clinic, i.e., images that are not high-quality scans and include distortions of various types. Despite the improvement provided by the signal decoder, the results were still suboptimal due to the limited training data available on photographed images. The network, which had been trained mainly on perfectly scanned images, became used to relying on ECG features extracted from the perfectly scanned images, features that would sometimes be obfuscated in the photographed images. To enhance performance on photos from the clinic, we employed a method from the area of transfer learning called domain adversarial training^14^. The network was augmented with a domain classifier that attempts to determine whether the input image is from the labeled NYU dataset (source domain) or the photographed images dataset (target domain). However, during training, this method backpropagates the gradients computed on the domain classifier through a gradient reversal layer, forcing the network training to choose domain agnostic features only. The result brings the performance on photographed images to be on par with that obtained on scanned images. The modified network underwent additional training using the photographed images dataset and using a fourth dataset consisting of a large set of unlabeled photographed images (DB4). The resulting trained neural network consisting of an encoder, a label predictor (LP), a signal decoder (SD), and a domain classifier (DC), was named ECG-AIO (ECG all-in-one).

To quantify the quality of our methods, we performed an experiment on photographed images taken in the clinic. The outcome was compared to that of a board-certified electrophysiologist. Finally, to demonstrate that the method is generic and can work with other tools as well, we modified the ECG-AIO network by replacing its encoder (feature extractor) network with ResNet18.

Figure 1 summarizes the general framework, network enhancements, experiments and results on the various datasets. Supplementary Fig. 2 further summarizes the progression from high-quality scans to photographed images.

Considering that the 12-lead ECG allows for classification of both morphological and arrhythmogenic diseases, the main body of this paper showcases examples of four prevalent rhythmical disorders (atrial fibrillation, atrial flutter, sinus bradycardia, sinus tachycardia), and common morphological conditions (left bundle branch block, left ventricular hypertrophy, premature ventricular contractions, right bundle branch block). These cardiac conditions were the most prevalent across all of our databases (DB1-DB3). Interpretability results for other cardiac conditions are presented in the supplement figure.

### Interpretability on labeled NYU dataset of high-quality scans

**Fig. 2:**
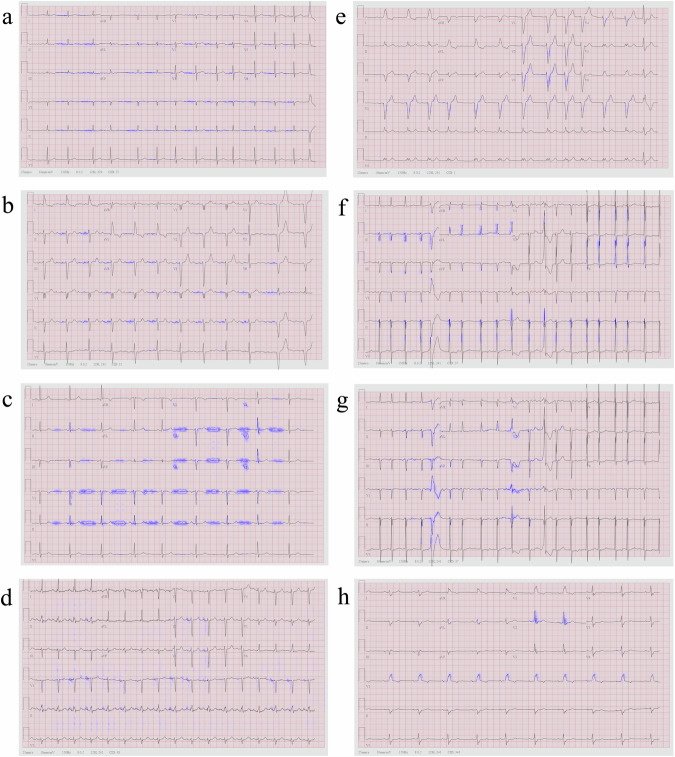
Interpretability performance of a neural network trained and tested on high-quality scans from the labeled NYU dataset. The interpretability marker is overlaid in purple on the original 12-lead ECG image captured from a patient with (**a**) atrial fibrillation, (**b**) atrial flutter, (**c**) sinus bradycardia, (**d**) sinus tachycardia, (**e**) left bundle branch block, (**f**) left ventricular hypertrophy, (**g**) premature ventricular contractions, or (**h**) right bundle branch block. The interpretability mechanism emphasized clinical features on the signal itself (without the background) indicative of each disease, without enforcing it algorithmically.

In this section, we showcase the interpretability results using a network that incorporates only the label predictor tested on the perfectly scanned labeled NYU dataset (DB1). Initially, we visualized the interpretability on the unseen (i.e., not used to train the system) labeled NYU dataset. Figure 2 provides a comprehensive illustration of the overall efficacy of the proposed interpretability method. The interpretability marker is overlaid in purple on the original 12-lead ECG image and highlights clinically pertinent features essential for diagnostic applications across various cardiac conditions. Notably, the interpretability mechanism excelled in emphasizing the signal while effectively ignoring the background. This nuanced approach ensures the clarity and relevance of clinically significant features without imposing unnecessary explicitness in the method, thereby ensuring its generalization across networks and ECG layouts.

The results underscore the interpretative capability of the system in capturing and highlighting key diagnostic elements in high-quality cardiac scans. Figure 2a shows an example of atrial fibrillation diagnosed in the clinic by the absence of P waves and the appearance of irregularly irregular rhythms. Indeed, our interpretability method accentuated the signal baseline surrounding the P wave location and annotated irregularly irregular rhythms through marked subsequent R peaks. Figure 2b shows an example of atrial flutter, diagnosed in the clinic by a “saw-tooth” pattern that replaced the P wave. The interpretability mechanism successfully underscored the “saw-tooth” pattern of the inverted flutter waves in leads II, III, aVF, loss of the isoelectric baseline, and upright flutter waves in V1, resembling P waves. Figure 2c shows an example of sinus bradycardia, clinically diagnosed by a heart rate below 60 bpm. Indeed, the interpretability mechanism emphasized the long flat periods between subsequent peaks, proportional to a low heart rate. Figure 2d shows an example of sinus tachycardia, clinically diagnosed by a heart rate above 100 bpm; the interpretability mechanism emphasized multiple subsequent peaks indicative of a heart rate above 100 bpm. Figure 2e shows an example of left bundle branch block, clinically diagnosed by prolonged QRS duration (usually exceeding 120 ms). The interpretability mechanism highlighted wide QRS complexes and other features that are used by physicians in the clinic, such as prolonged QRS duration (usually exceeding 120 ms) and prolonged R wave peak time (longer than 60 ms in leads V5-6), along with the absence of Q waves in lateral leads. Figure 2f shows an example of left ventricular hypertrophy, clinically diagnosed by R wave in V4, V5, or V6 wider than 26 mm, along with non-voltage criteria, e.g., increased R peak time, ST segment depression and T wave inversion in left-sided leads, indicative of left ventricular ‘strain’ pattern. The interpretability mechanism marked R wave peaks in V4, V5, V6 and inverted T wave on this ECG record. Figure 2g shows an example of premature ventricular contractions, clinically diagnosed by a broad QRS complex (above 120 ms) with abnormal morphology. Indeed, the interpretability mechanism detected and marked discordant ST segment and T wave changes for the right complex. Figure 2h shows an example of right bundle branch block, clinically characterized by “M-shaped” QRS complexes (referred as RSR’ patterns), wide slurred S waves in lateral leads, and numerous complexes with QRS duration longer than 120 ms. All these features were precisely detected by the interpretability mechanism.

### Interpretability on labeled NYU dataset with shadows and artifacts

**Fig. 3:**
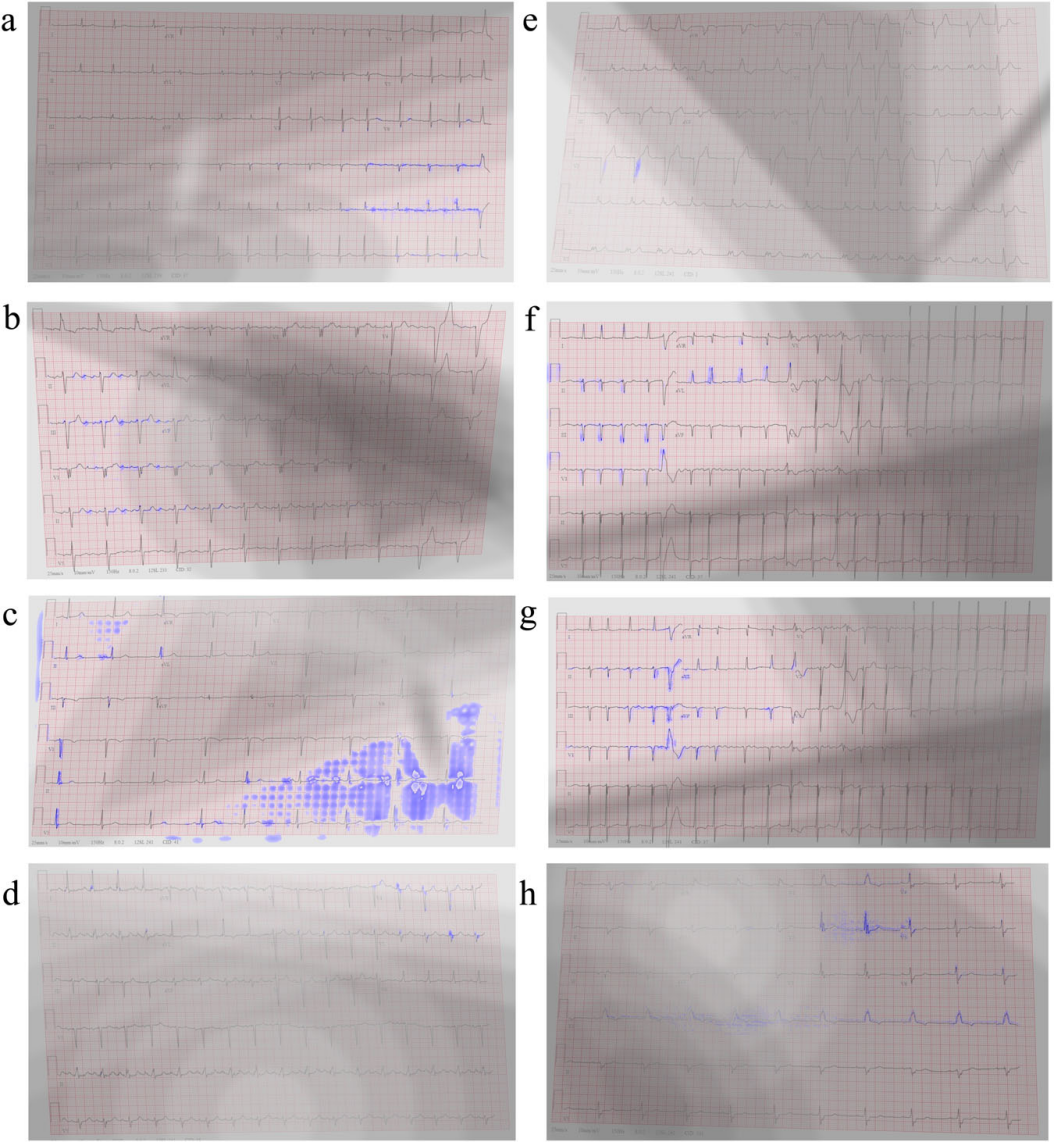
Interpretability performance of a neural network trained on the labeled NYU dataset and tested on the same dataset following addition of shadows and artifacts. The interpretability marker is overlaid in purple on the original 12-lead ECG images collected from a patient with (**a**) atrial fibrillation, (**b**) atrial flutter, (**c**) sinus bradycardia, (**d**) sinus tachycardia, (**e**) left bundle branch block, (**f**) left ventricular hypertrophy, (**g**) premature ventricular contractions, or (**h**) right bundle branch block. In most cases, the interpretability ignored the shadows of the image. In some other cardiac conditions, e.g., right bundle branch block, the interpretability was incorrect, causing also lack of resolution and shifting of the signal out of focus.

Figure 3 shows performance of the interpretability mechanism on the labeled NYU dataset with shadows and artifacts (DB2) superimposed on the same images from Fig. 2. In most cases, the interpretability ignored the shadows on the images. In some other cases, like right bundle branch block, the interpretability was incorrect. One limitation of our interpretability method is that it is heavily dependent on the classification accuracy, which, in turn, relies on the sample being from the same distribution as the training set. The introduction of occlusion artifacts negatively impacted both the network and the interpretability algorithm accuracy, as depicted in Fig. 3.

**Fig. 4:**
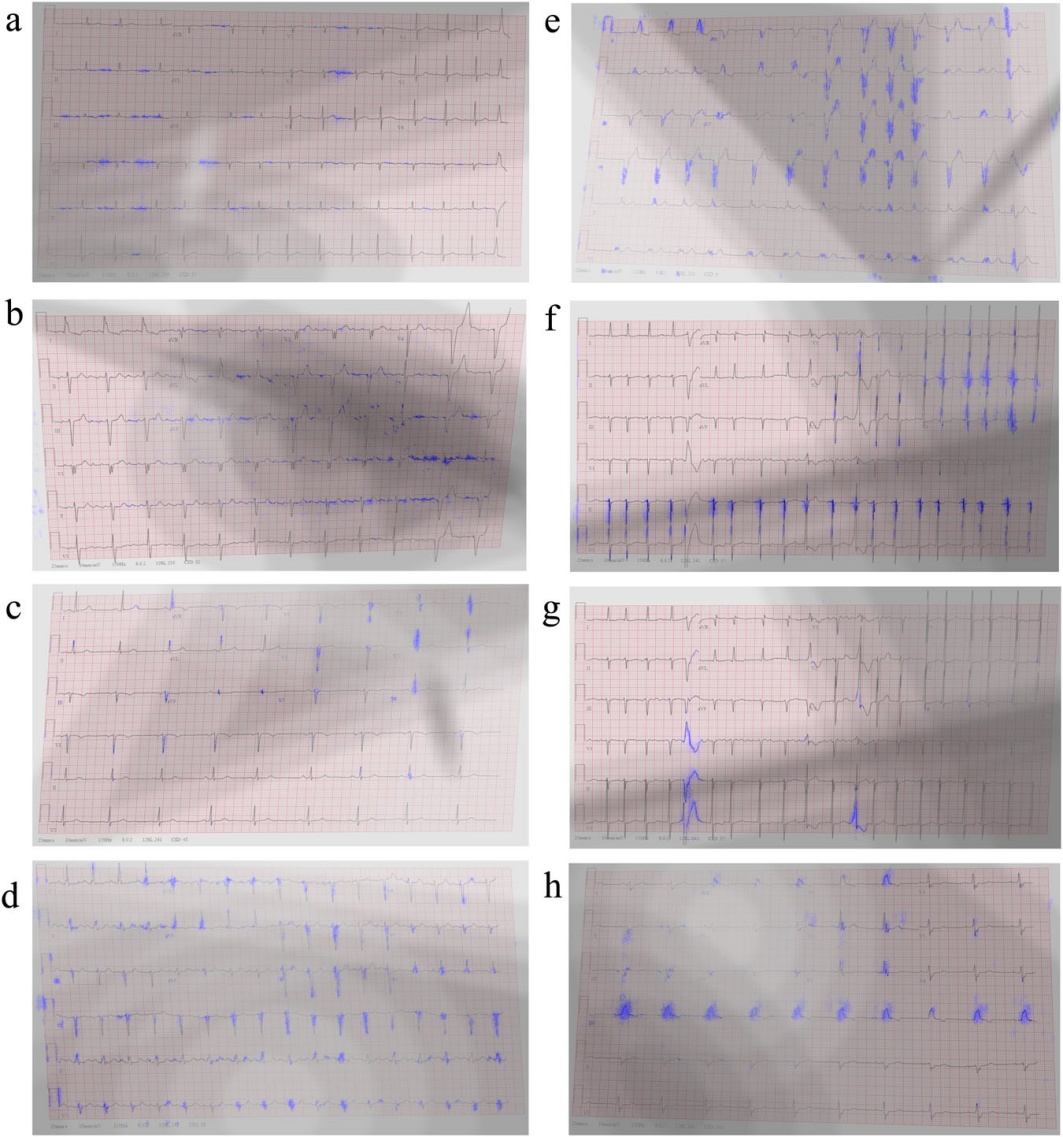
Interpretability performance of a neural network trained and tested on labeled NYU dataset with shadows and artifacts (DB2). The interpretability marker is overlaid in purple on the original 12-lead ECG image collected from a patient with (**a**) atrial fibrillation, (**b**) atrial flutter, (**c**) sinus bradycardia, (**d**) sinus tachycardia, (**e**) left bundle branch block, (**f**) left ventricular hypertrophy, (**g**) premature ventricular contractions, or (**h**) right bundle branch block. The interpretability mechanism emphasized clinical features on the signal itself (not in the background) that were relevant for each disease, without enforcing it algorithmically.

To demonstrate the generalizability and robustness of our proposed interpretability method and to ascertain whether the shortcomings were attributable to the classifier network vs. the interpretability method, we augmented the network by incorporating a signal decoder whose task is to reconstruct the image, thus compelling the network to consider both bright and dark (shadowed) areas of the image. The effectiveness of the resulting network on the NYU dataset with shadows and artifacts (DB2) is presented in Fig. 4. The figure shows that clinically relevant features crucial for detection of specific cardiac conditions were detected even in heavily saturated images. In the case of atrial fibrillation (Fig. 4a), the method continued to highlight the signal baseline surrounding the P-wave location, emphasizing the absence of P-waves, and annotating irregularly irregular rhythms through marked subsequent R-peaks, both in well-lit and shadowed regions of the image. For atrial flutter (Fig. 4b), the interpretability mechanism underscored the “saw-tooth” pattern of inverted flutter waves in leads II, III, aVF, located in the more illuminated part of the image, and the loss of the isoelectric baseline on the right side of the darker region. For sinus bradycardia (Fig. 4c), unlike the previous experiment (Fig. 3c) where long flat periods between subsequent peaks were marked, the subsequent peaks were now highlighted, providing one possible criterion for diagnosing the condition. For sinus tachycardia (Fig. 4d), multiple subsequent peaks were still accentuated, resulting in a heart rate exceeding 100 bpm. Regarding left bundle branch block (Fig. 4e), the algorithm still accentuated the majority of wide QRS complexes, meeting clinical criteria for prolonged QRS duration and prolonged R-wave peak time, along with the absence of Q waves in lateral leads. In the case of left ventricular hypertrophy (Fig. 4f), the modified algorithm still emphasized the high R-wave peaks in V4, V5, V6, meeting the criteria of the target disease. Additional conditions, such as ST segment depression and T-wave inversion in left-sided leads, were also highlighted in Fig. 4f. For premature ventricular contractions (Fig. 4g), the relevant complexes were once again detected, with an emphasis on discordant ST segment and T-wave changes for the right complex. In the case of right bundle branch block (Fig. 4h), numerous “M-shaped” QRS complexes (referred to as RSR patterns) were highlighted, along with wide slurred S waves in lateral leads. Additionally, many complexes with QRS duration greater than 120 ms were emphasized. Note that the interpretability method (Jacobian) remained unchanged; only the network was improved.

### Interpretability on photographed images taken by a smartphone in the clinic

**Fig. 5:**
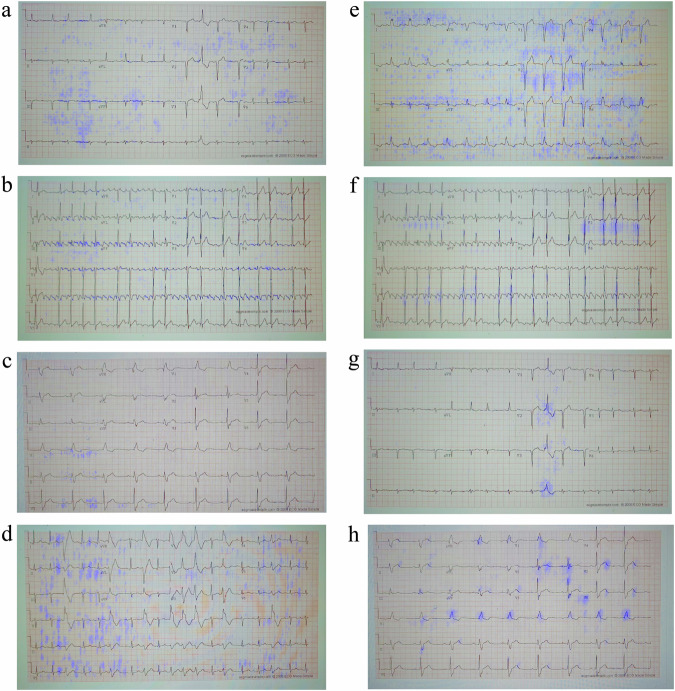
Interpretability results of a neural network trained and tested on labeled NYU dataset with shadows and artifacts and tested on photographed images from the clinic (DB3). The interpretability marker is overlaid in purple on the original 12-lead ECG image collected from a patient with (**a**) atrial fibrillation, (**b**) atrial flutter, (**c**) sinus bradycardia, (**d**) sinus tachycardia, (**e**) left bundle branch block, (**f**) left ventricular hypertrophy, (**g**) premature ventricular contractions, or (**h**) right bundle branch block. The interpretability lacked resolution and did not adequately focus on the signal. In many cases, such as A, D, E, F and H, a substantial part of the interpretability marker appeared on the background rather than on the signal.

The performance of our approach on photographed images (DB3) was then considered using a network that incorporated an encoder and a label predictor only. Figure 5 shows the results on mobile-captured clinical images from patients with common rhythmic morphologies (atrial fibrillation (Fig. 5a), atrial flutter (Fig. 5b), sinus bradycardia (Fig. 5c), sinus tachycardia (Fig. 5d)) and common morphologies (left bundle branch block (Fig. 5e), left ventricular hypertrophy (Fig. 5f), premature ventricular contractions (Fig. 5g), right bundle branch block (Fig. 5h)).

As can be seen in Fig. 5, the interpretability still exhibited certain ambiguities when applied to photographed images. We attribute these inaccuracies to artifacts which weren’t imitated by the augmentation that we applied, and to variations in formatting and layout. Furthermore, in specific instances, such as with the left bundle branch block image, certain artifacts appeared on the background of the checkered ECG paper.

**Fig. 6:**
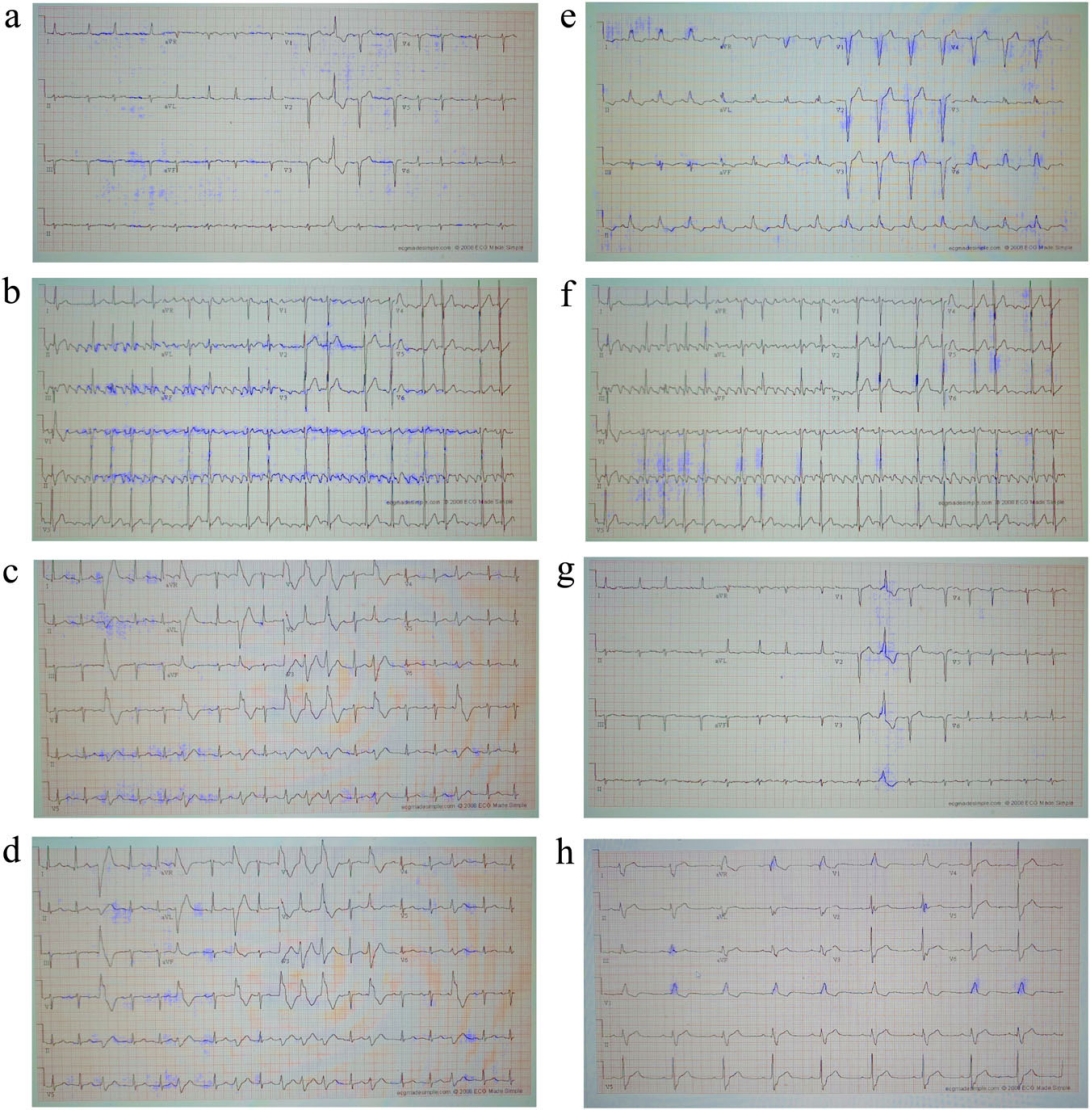
Interpretability performance of a neural network trained on photographed images (DB3) from the labeled NYU dataset with shadows and artifacts (DB2) and on unlabeled mobile-captured images from the adversarial database (DB4) and tested on photographed images. The interpretability marker is overlaid in purple on the original 12-lead ECG image collected from a patient with (**a**) atrial fibrillation, (**b**) atrial flutter, (**c**) sinus bradycardia, (**d**) sinus tachycardia, (**e**) left bundle branch block, (**f**) left ventricular hypertrophy, (**g**) premature ventricular contractions, or (**h**) right bundle branch block. The interpretability method emphasized clinical features on the signal itself (avoiding the background) that were relevant to each disease, without enforcing it with a specific algorithm.

To address the problem, the network was enhanced with a signal decoder and a domain classifier, were the resulting architecture is called ECG-AIO (ECG all-in-one). In addition, ECG-AIO was subjected to a special training procedure, called domain adversarial training, taken from the field of transfer learning^14^ (see method section). Training used the labeled NYU images with and without superimposed shadows and artifacts, a limited set of photographed labeled 12-lead ECG images in various formats, and a substantial unlabeled dataset of photographed 12-lead ECG images (DB4). After this training, the interpretability performance on the same untrained images in Fig. 5 show increased robustness and clarity, as illustrated in Fig. 6. In the case of atrial fibrillation (Fig. 6a), the signal baseline surrounding the P-wave location was emphasized, highlighting the absence of P waves and annotating irregularly irregular rhythms through marked subsequent R peaks. For atrial flutter (Fig. 6b), the interpretability mechanism underscored the “saw-tooth” pattern of flutter waves present in many signals. Sinus bradycardia (Fig. 6c) was characterized by numerous subsequent QRS complexes, indicative of slow cardiac activity without other morphological abnormalities. Sinus tachycardia (Fig. 6d) was distinguished by periods between subsequent QRS complexes, indicative of a fast heart rate. In the case of left bundle branch block (Fig. 6e), most of the signal was accentuated, aligning with the diagnostic criterion of prolonged QRS duration (longer than 120 ms), thus emphasizing numerous long QRS complexes. For left ventricular hypertrophy (Fig. 6f), the algorithm emphasized the high R-wave peaks in V4, V5, V6, meeting the disease criteria, and highlighted ST segment depression and T wave inversion in left-sided leads. Premature ventricular contractions (Fig. 6g) were identified by highlighting the relevant complexes. In the record of a right bundle branch block (Fig. 6h), almost all QRS complexes were highlighted due to the diagnostic criteria of prolonged QRS (longer than 120 ms) and a M-shaped QRS complex pattern in V1-3 leads. In addition, slurred wide S waves following the wide QRS complexes were emphasized.

The high interpretability was accompanied by strong classification accuracy (see Supplementary Table 1).

To further demonstrate the robustness of our method, we selected 200 samples of atrial flutter (rhythmic) and 200 samples of premature ventricular contraction (morphological) and defined a result as a true positive (TP) if 90% or more of the network signal features colocalized with known relevant clinical features of the specific cardiac condition. Conversely, if 90% or more of the network signal features did not localize with relevant clinical features of a specific cardiac condition, we defined it as a true negative (TN). Ideally, the same sample should qualify as both TP and TN. We defined a result as a false positive (FP) if less than 90% of the network signal features colocalized with relevant clinical features, and as a false negative (FN) if 10% or more of the network signal features localized with irrelevant clinical features of the specific cardiac condition. Supplementary Fig. 3 shows the confusion matrix of this experiment.

To illustrate that the presence of off-axis and rotated images does not affect the quality of our results, we selected 5 heavily distorted clinical images (Supplementary Fig. 4) for each clinical condition and compared them to the confusion matrix of random images mentioned above. No significant differences in performance were found.

### Interpretability of absence of specific cardiac condition

**Fig. 7:**
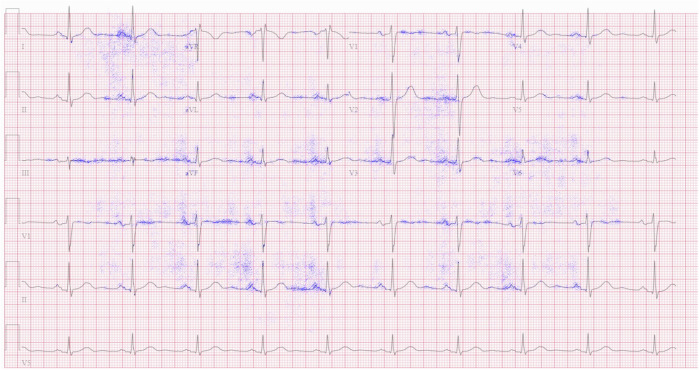
12-lead ECG image demonstrating the absence of atrial fibrillation. The interpretability mechanism correctly highlighted the presence of a P wave.

Our interpretability method allows us to quantify how much each pixel in the input image contributes to the final decision. Thus, we can generate an interpretability image not only for the presence but also for the absence of a cardiac condition. This process allows us to gain insights into the specific areas within the ECG image that the model considers important to rule out a certain cardiac condition. Fig. 7 shows an example of a record without signs of an atrial fibrillation. The interpretability model emphasized the presence of a P wave before each QRS complex, confirming the absence of atrial fibrillation.

### Generality of the proposed interpretability tool

**Fig. 8:**
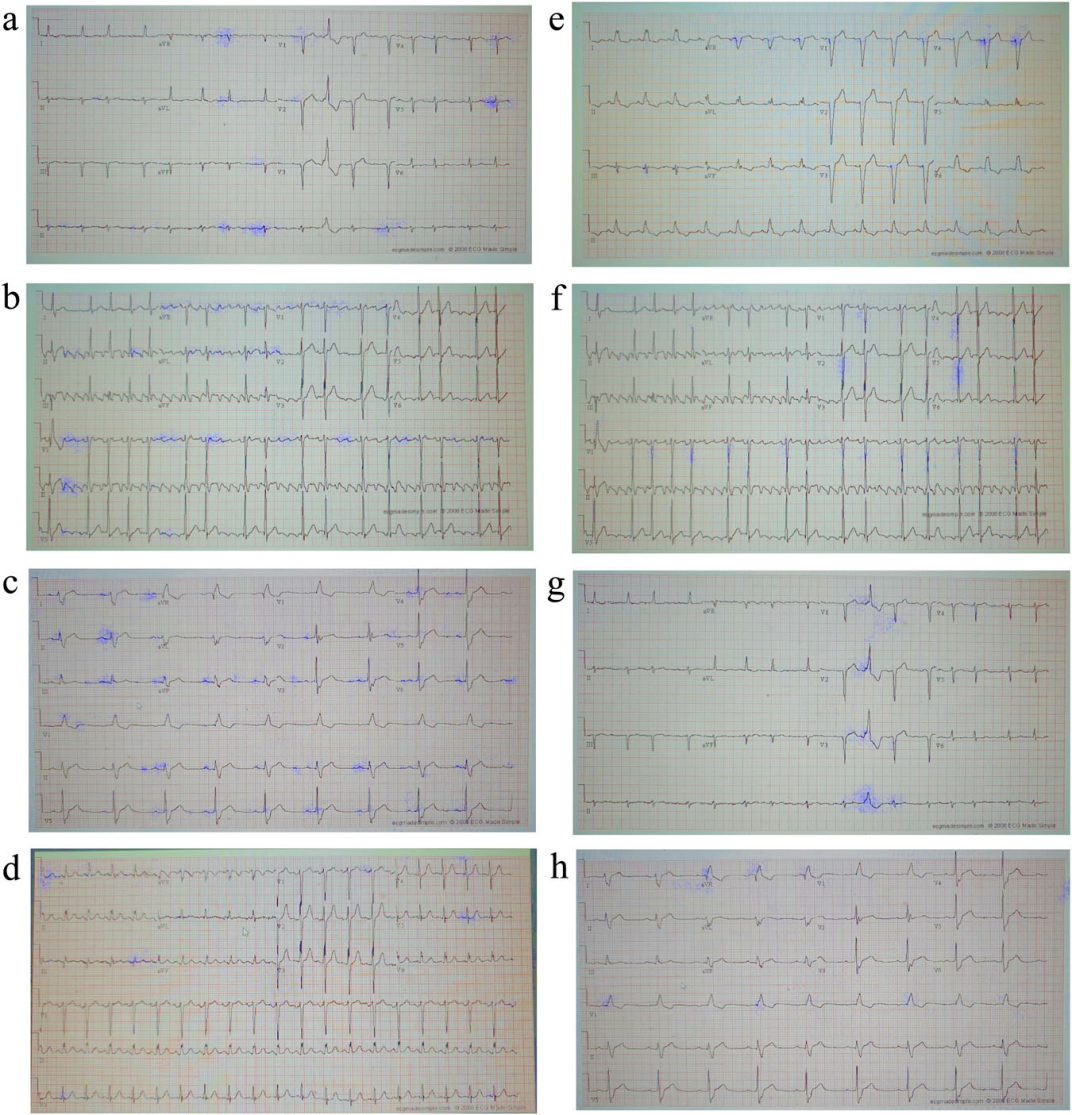
Interpretability performance of ECG-AIO with an encoder based on ResNet18. The network was trained on photographed images, the NYU dataset with shadows and artifacts, and unlabeled photographed images from the adversarial database. The network was tested on photographed images. The interpretability marker is overlaid in purple on the original 12-lead ECG image collected from a patient with (**a**) atrial fibrillation, (**b**) atrial flutter, (**c**) sinus bradycardia, (**d**) sinus tachycardia, (**e**) left bundle branch block, (**f**) left ventricular hypertrophy, (**g**) premature ventricular contractions, or (**h**) right bundle branch block. The interpretability mechanism emphasized clinical features on the signal itself (without the background) that were relevant for each disease, without enforcing it algorithmically.

To demonstrate that our approach is generic across network architectures, we replaced the feature extractor in the ECG-Adversarial network with ResNet18^1^ and tested it on the same records as in Fig. 6. As illustrated in Fig. 8, the highlighted features were similar. Indeed, in all experiments, the correctness of the approach required no specific treatment by the algorithm while addressing different features, different diseases, different layouts, different networks, or other.

Next, we used ECG-AIO with the ResNet18-based encoder to demonstrate interpretability on additional cardiac conditions. Supplementary Fig. 5 illustrates records of diverse cardiac conditions, such as first-degree AV block^15^, myocardial infarction^16^, pacing^17^, short PR interval, ST elevation, and Wolff-Parkinson-White syndrome. In the instance of first-degree AV block, emphasis was placed on the prolonged PR interval, a central criterion for detecting this cardiac disorder. For myocardial infarction, the algorithm highlighted ST-segment elevation in leads I, aVL and V1-V5. Pacing was discerned through prominently emphasized pacing impulses. In cases of a short PR interval, the algorithm directs attention to the PR intervals. Regarding ST elevation, the algorithm accentuated the elevated ST segment, while for Wolff-Parkinson-White Syndrome, it underscored a short PR interval and a delta wave, the most common feature for identification of the syndrome^18^. Compared to Supplementary Fig. 1 (GRAD-CAM interpretability), our algorithm generated highly specific and high-resolution outputs, specifically focusing on delta waves while neglecting the background. In contrast to GRAD-CAM, for Wolff-Parkinson-White Syndrome (Supplementary Fig. 1B), our method pointed to the relevant clinical features only, namely, a short PR interval and a delta wave.

### Clinical assessment of interpretability using several layouts

To evaluate the robustness of interpretability across different formats, ECG records of premature ventricular contraction collected in different formats were analyzed. Supplementary Fig. 6 illustrates consistent highlighting of relevant clinical features in all examples, regardless of the layout that was used to draw the leads of the ECG. Importantly, the algorithm did not detect the format nor enforce any format-specific operations to identify the features.

To compare the performance of our method vs. GRAD-CAM, we utilized published 12-lead ECG records with superimposed heatmaps generated by GRAD-CAM^12^. After removing all markups, the cleaned images were used as input for our algorithm. The results are displayed in Supplementary Fig. 7. Note the clear features highlighted by our method compared to the heatmap generated by the GRAD-CAM.

### Interpretability on 12-lead ECG signals

To demonstrate the robustness of interpretability across different input formats, we rendered the signal on a 12-lead ECG image and analyzed it. The results are presented in Supplementary Fig. 8. This figure shows two records from the China 12-lead ECG Challenge database^19^. Both records, which contain premature ventricular contractions, illustrate that our algorithm performs effectively on rendered digital vectors.

### Clinical assessment of the interpretability accuracy

**Fig. 9:**
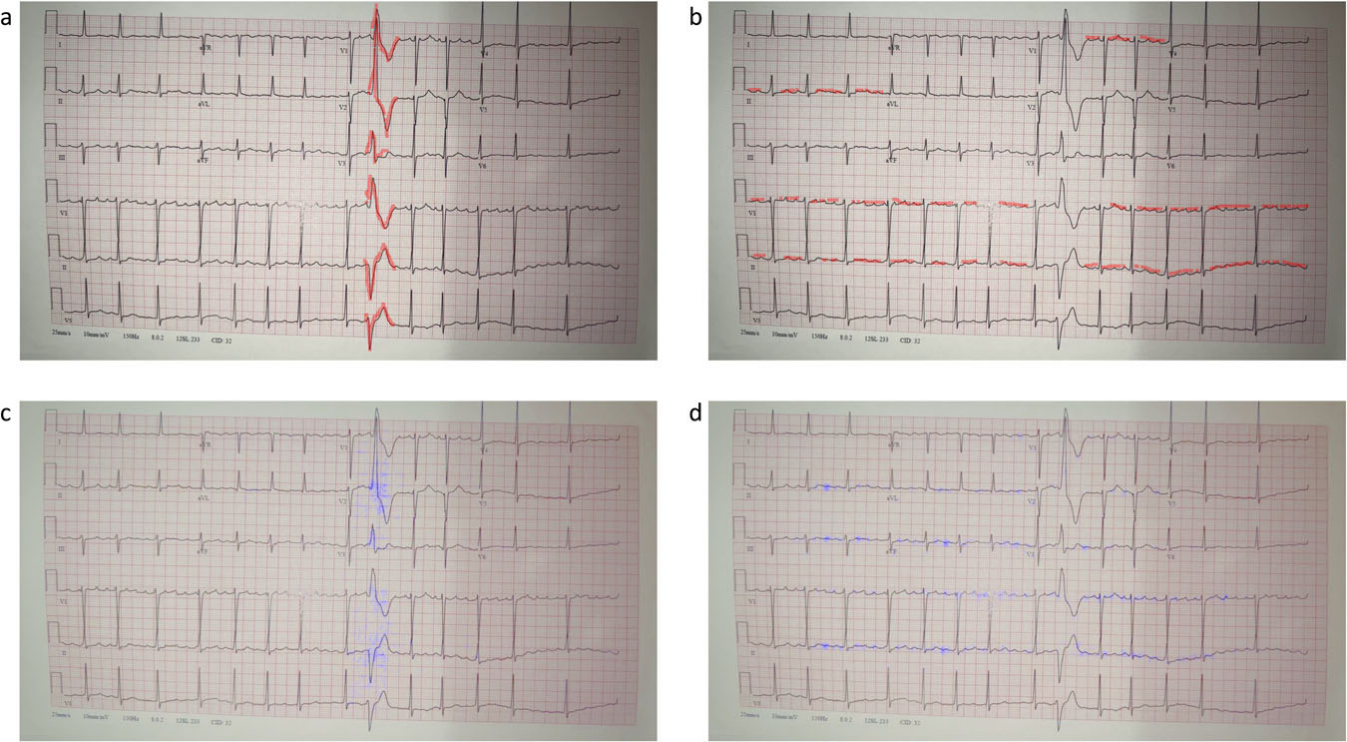
Interpretability comparison between cardiologists and our algorithm. Interpretability comparison between cardiologists (red marking) and the algorithm (purple marking) for an image captured in the clinic featuring both atrial flutter (AFL) and premature ventricular contraction (PVC). The figure shows an ECG record of a patient exhibiting both conditions, and the interpretability output provided by the algorithm and by a cardiologist. **a** Cardiologist-identified features crucial for the detection of PVC. **b** Cardiologist-identified features crucial for the detection of AFL. **c** PVC features identified by the algorithm. **d** AFL features identified by the algorithm.

To evaluate the clinical performance of our methods, we conducted a comparison between the interpretability achieved by our algorithm and that achieved by 3 electrophysiologists, focusing on two cardiac conditions—atrial flutter, which is an arrhythmogenic condition and a morphological condition - premature ventricular contractions. A representative example showcasing electrophysiologist interpretability superimposed over our interpretability is presented in Fig. 9. The quantitative measure used to evaluate the correlation between the algorithm results and the interpretations provided by electrophysiologists is the correlation coefficient (see Methods section). The mean coefficient for images associated with premature ventricular contractions was 0.94 ± 0.03. The mean coefficient for atrial flutter images was 0.96 ± 0.1. The high (close to 1) values suggest the strong agreement and reliability of our method in capturing and interpreting features associated with these cardiac conditions and typically manually identified by experts.

## Discussion

Interpretability of an AI system is a crucial factor for gaining acceptance and trust from physicians, particularly in the case of a 12-lead ECG classifier. However, interpretability methods suitable for photographed images taken in clinical practice are currently lacking. This study demonstrated the effectiveness of a general approach in providing interpretability, measured through features it highlighted in the ECG trace. The proposed generic deep network offers interpretability for both morphological and arrhythmogenic cardiac conditions in photographed images. Furthermore, it enables insights into specific areas within the ECG image that the model deems significant for ruling out particular cardiac conditions.

For the first time, we introduce interpretability for images photographed by mobile camera. In numerous clinics worldwide, ECG recordings are either provided as printed paper or electronically in image format. Given that in numerous healthcare environments, limited resources prevent the storage of digital waveform signals from ECGs, interpretability algorithms designed to only handle digitally formatted signals, rather than printouts or digital images, lack clinical relevance. When ECG recordings are presented on printed paper, the digitization is often achieved using a smartphone camera. However, images captured with a mobile camera frequently exhibit various artifacts, are tilted, skewed, crumpled, and may include shadowing. Our method excels in providing interpretability even for the skewed and crumpled segments of the image and is therefore of value in clinical practice.

We demonstrated the ability of the proposed approach not only to emphasize features indicative of specific cardiac conditions, but also to highlight features that conclusively demonstrate the absence of such conditions. Thus, the unique strength of our interpretability method empowers physicians by providing them with valuable information regarding the absence of such conditions, thereby heightening confidence and acceptance of the approach within the clinical team. This not only contributes to a more comprehensive understanding of the diagnostic landscape but also plays a pivotal role in early detection and correction of misdiagnoses, ultimately improving the overall reliability and efficacy of cardiac assessments.

Interpretability techniques can be broadly categorized into model-specific vs. model-agnostic methods^20^, in which the model is the learnt classifier. Opting for a model-agnostic approach, we leveraged techniques that offer interpretability independent of internal model parameters. Remarkably, when retraining our network —such as transitioning from photographed images to scanned images with shadows — no adjustments were necessary, thereby proving the adaptability of our chosen model-agnostic method. Although model-agnostic techniques are often criticized for providing limited insights, our study demonstrated their utility in gaining a valuable understanding of model performance. For instance, in the original model, shadows were disregarded. However, by compelling the model to retrain on shadow-inclusive data, we witnessed a marked improvement in its performance.

Quantifying interpretability performance becomes a challenge when numerous low-resolution features are present. To address this, we developed a novel metric-based method which compared the output of our system with that drawn by physicians. This approach offers a more robust and quantitative evaluation of interpretability performance in the presence of intricate, low-resolution features.

Post-hoc interpretability, the foundation of our approach, treats the model as a complex black box after training. This stands in contrast to intrinsic interpretability, where interpretability is an integral aspect of the model design, allowing a deeper understanding of its inner workings. The notable advantage of post-hoc interpretability is its generic applicability, as it doesn’t require specific training for each network. This versatility contributes to its broader usability across various machine learning models. In addition, computing the Jacobian matrix is resource-intensive, as it involves calculation of the partial derivatives of the output with respect to each input dimension. This can scale poorly with larger models, especially when dealing with high-dimensional inputs. However, it is important to note that these partial derivatives are already calculated during forward propagation and stored within the computational graph. Therefore, their multiplication to compute the Jacobian is relatively straightforward. Importantly, our interpretability method can seamlessly extend to other networks, such as ResNet18, without requiring any adaptation. Thus, our interpretability method can be used for other medical modalities such as MRI and CT images.

Previous efforts have been made to generate ECG interpretability based on one-lead ECG (refer to^9^ for a review). However, interpretability derived from one-lead ECG (as seen in, for example^21^) is limited in its ability to detect most morphological conditions, and primarily focuses on arrhythmogenic aspects. Additionally, one-lead ECG data is typically available as a signal rather than as an image. Consequently, this raises challenges in capturing the full spectrum of morphological intricacies. Our approach encompasses a broader scope by detecting both morphological conditions and arrhythmogenic patterns. Additionally, our method is designed to handle the intricacies of ECG data presented as images rather than signals. This versatility not only enhances the comprehensiveness of interpretability but also aligns with the practicality of real-world clinical scenarios, where 12-lead ECG data is often available in image format. By addressing these challenges, our interpretability framework aims to provide a more holistic understanding of 12-lead ECG patterns for improved clinical insights.

Granular interpretability is likely the most reliable method for identifying a wide range of cardiac disorders. While some conditions, such as premature ventricular contractions, are easily recognizable on an ECG snapshot, others, like cardiac amyloidosis, are far more challenging to diagnose. Cardiac amyloidosis is a rare condition caused by the buildup of a harmful substance called amyloid in the extracellular space^22^. In a 12-lead ECG record, it may present as a pseudo-infarction pattern, with low voltages in the limb leads and Q waves in the anterior and inferior leads. High-resolution and granular interpretability are crucial for detecting such subtle electrophysiological signs. ECG readings in patients with cardiac amyloidosis may also include varying degrees of AV block, with detection depending heavily on P-R intervals. In such cases, identification of tiny P waves, which may span only a few pixels on an ECG snapshot, essential^23^.

Ayano et al.^9^, in their review, discuss various methods for interpreting models applied to ECG data. While the interpretability of 12-lead ECG is a critical clinical step, their review does not address the robustness of these methods against image artifacts, such as noise and natural limitations like occlusions, shadows, or crumpled paper, which are commonly encountered in clinical settings. Among the approaches outlined in their review, our method aligns most closely with ‘saliency maps,’^24^ which leverage the gradients of an machine learning model’s output with respect to its input. However, unlike saliency maps, which approximate the network’s output using a first-order Taylor expansion, our method employs complete gradient maps calculated through the chain rule. Additionally, as noted by Ayano et al.^9^, gradients are often noisy and unstable. Their method was not applied to images affected by natural artifacts and noise. In contrast, we present a more accurate gradient mapping approach that performs well on both perfectly scanned images and those impacted by noise and distortions.

Image denoising techniques are widely recognized and have been extensively discussed by Jin et al.^25^. Many of these methods are already integrated into high-end mobile devices. However, capturing 12-lead ECG printouts presents unique challenges that existing mobile device algorithms are not designed to handle. These challenges include occlusion artifacts, shadowing, handwritten text, crumpled paper, and aliasing caused by subsampling the background grid. This paper addresses these issues and demonstrates effective solutions to overcome them.

The study has two main clinical impacts: (i) One of the main limitations of using AI in clinical environments is the lack of interpretability. Improving interpretability can enhance physician trust in AI systems. For example, an AI system that automatically diagnoses a 12-lead ECG can be particularly helpful when a physician is unfamiliar with certain cardiac conditions and their associated features. Such a system can also support physicians without specialized training in cardiology. Additionally, clinical features are sometimes overlooked, and interpretability provides feedback that can improve physician accuracy and patient outcomes. (ii) A key challenge in integrating 12-lead ECG AI systems into real clinical environments is the variety of ECG layout formats and the absence of a global protocol or standard for 12-lead ECG machines. Additionally, many 12-lead ECG devices do not support data export in specific formats. Capturing the ECG output via a smartphone circumvents this compatibility issue, providing a universal method for acquisition, classification and interpretation across different clinical settings.

The main limitation associated with our approach is its exclusive reliance on partial derivatives per pixel, overlooking the inherent characteristics of the ECG pattern and the intricate interrelationships between adjacent pixels. Consequently, the interpretability output may exhibit a certain level of granularity, as evident in the provided figures, particularly Fig. 6. Additionally, the described interpretability method may, on occasion, selectively emphasize only a limited segment of the overall phenomenon crucial for diagnosis. For example, in cases involving premature ventricular contractions, the method may exclusively highlight a subset of premature complexes present in the 12-lead ECG image. Another noteworthy limitation is the direct correlation between the accuracy of the interpretability method and the network resolution, which showed a proportional relationship. In particular, for convolution kernels, the method encounters constraints in achieving a resolution significantly higher than that of the network kernel. This inherent limitation can impede the ability to provide detailed interpretability, especially when attempting to elucidate intricate features beyond the scale defined by the network’s convolutional kernels. Another primary limitation pertains to the fact that the Jacobian matrix consists of partial derivatives of the output. As a result, it is inherently suited only for networks without memory, where any change in input is immediately reflected in the output. Networks with memory, such as long short-term memory networks (LSTMs), recurrent neural networks (RNNs), transformers, and gated recurrent units (GRUs), are therefore incompatible with the proposed method. Moreover, for some cardiac conditions, there are several criteria used for diagnosis. The clinical properties the network selects for detection cannot be controlled, which, in turn, affects interpretability. Additionally, as demonstrated in this study, the network currently shows high accuracy and demonstrates the feasibility of a generic, clinical resource interpretability tool for AI models analyzing 12-lead ECG images. However, this alone is not sufficient to determine a comprehensive clinical diagnosis. Additional information from medical records, other diagnostic data and physician expertise is required. The primary purpose of the tool is to assist physicians in identifying the presence or absence of specific clinical conditions. Furthermore, as with any AI system, insufficient sampling of diverse patient populations or limited data for rare cardiac conditions can negatively impact the system’s accuracy and, consequently, its interpretability. Finally, while AI offers efficiency and diagnostic support, and can even enhance treatment personalization, over-reliance on AI can erode essential clinician expertise. This concern does not only encompass the risk for occasional errors, but extends to clinician dependence on its recommendations, potentially losing critical skills and clinical intuition over time.

Several future directions may be explored. The interpretability accuracy is heavily dependent on the accuracy of the network itself. With increasing exposure to data, the network accuracy will improve, and as a direct result, its interpretability accuracy will also increase. Moreover, a smartphone-based ECG interpretability tool has the potential to redefine telemedicine and remote ECG interpretation, by enhancing accessibility to and efficiency of cardiac monitoring. However, to realize its full potential, developers and healthcare providers must address challenges related to diagnostic accuracy, regulatory compliance, data security and patient education. Future clinical smartphone-based ECG application will include four steps. The clinician will receive a 12-lead ECG plot, either as a hardcopy or displayed on a computer screen. Using a dedicated mobile application, the clinician will take a photo of the 12-lead ECG plot, which will be processed by the application processes and a list of possible diagnoses will be returned. The clinician will then review each diagnosis by examining the interpretability provided by the application, and then confirm or reject the diagnosis based on the patient’s medical file and additional clinical data. In addition, certain treatments are not definitive, and cardiac conditions may recur, such as in cases of cardiac resynchronization therapy or atrial fibrillation after RF ablation. By automatically diagnosing the 12-lead ECG and marking relevant features, AI can enable physicians to remotely monitor the recurrence of cardiac disorders. Finally, future clinical research should evaluate the impact of an interpretability tool on the diagnostic process in real-world scenarios. For example, in a comparative study, one group of physicians can be provided an AI diagnosis without access to the interpretability tool, while the another group is allowed access to both the AI diagnosis and the interpretability tool after making an initial decision. Both groups would then assess the AI’s recommendations, and the level of agreement with the gold standard diagnosis can be determined. Additionally, the study can analyze whether there was a significant number of changes in the physicians’ decisions in the group with access to the interpretability that improved accuracy.

## Methods

### Datasets

#### DB1: Labeled NYU dataset of high-quality scans

The publicly accessible NYU ECG Database (https://education.med.nyu.edu/ecg-database/app), initially composed of 98,420 resting 12-lead ECG scanned images, is sorted into 93 possible cardiac condition categories. An individual ECG can indicate multiple cardiac conditions, leading to multi-label scenarios in certain instances. The database was limited to ECGs from patients aged 18-80 years, resulting in 81,287 ECGs. Unfortunately, 2,061 ECG images were unattainable for download; hence, the final NYU dataset includes 79,226 scanned ECG images.

Each scanned image was associated with a clinical diagnosis provided by a cardiologist. Despite the broad range of diagnoses, some diagnostic categories were underrepresented. As deep learning methods require a large dataset, we assembled several subgroups. For instance, we assembled ‘right bundle branch block (RBBBs)’ together with ‘bundle-branch block - RBBB – incomplete’ and ‘bundle branch block - right – RBBB’. The number of positive and negative samples for each category is presented in Supplementary Table 2.

After an initial split of the data into development (data used for training the model, 85% of the population; 67,351 records) and holdout (data used to test the system accuracy, 15% of the population; 11,885 records) datasets, the development dataset was further divided into training (94% of the development set) and internal validation (6%) datasets.

#### DB2: Labeled NYU dataset of high-quality scans with superimposed shadows and artifact

To address artifacts in mobile-captured ECGs, such as random shadows and varying image perspectives, we implemented a comprehensive domain adaptation strategy. The neural network processes both high-quality scans and photographed images alternately to ensure robust performance across diverse image sources. Upon examination of the interpretability output of the model, we observed that it learned to disregard certain domain-specific features, particularly shadows (see Fig. 3). As this can potentially lead to the underdiagnosis of cardiac conditions, we developed a comprehensive augmentation strategy for the entire set of high-quality scanned ECG images (DB1). This strategy incorporated two main components: perspective modifications (changing the angle from which a mobile device might have captured the image) and the introduction of artificial shadows. Additionally, we included extra illumination sources to simulate image saturation resulting from flash usage.

Synthetic shadows, mimicking diverse lighting conditions encountered in real-world environments, were added to each image. Four shadows were incorporated into each image, with their intensities chosen randomly from a uniform distribution between 0.3 and 0.7 of the maximum color intensities. This approach allowed us to replicate a broad array of possible shadow situations. In addition, the shadows were incorporated in a variety of shapes, including polygons, ellipses, and single source shadows, resulting in a large selection of light and shadow effects. This strategic diversity ensured model exposure to an extensive range of shadow types and intensities during the training phase, enhancing its capability to handle variations in photographed images. Simultaneously, we implemented a dynamic blurring effect dependent on the shadow intensity, mimicking the shadowing effects of objects in clinical practice. For examples before and after the artificial augmentation, refer to Fig. 3.

#### DB3: Mobile device-acquired labeled 12-lead ECG images of various formats

To simulate real-life clinical practice, we gathered 1807 labeled 12-lead ECG images from ‘Dr. Smith’s ECG Blog’ (https://hqmeded-ecg.blogspot.com/) and ‘ECG Made Simple’ (https://ecgmadesimple.ca/) blogs. These images were printed and then captured by either Android or iPhone cell phones. This diversified dataset provides a more realistic representation of the clinical practice data that our model will encounter, enhancing its ability to generalize to lower-quality, highly variable images. The images were randomly selected from websites, and none were excluded. Full HD resolution was used to capture snapshots with mobile cameras. This resolution was chosen to ensure that even the most delicate features, such as tiny P waves, would occupy several pixels, especially when the entire ECG page fills most of the frame. The blur detection algorithm of OPENCV library was used^26^. This algorithm calculates the variance of the Laplacian of the image, and if the image had a variance of Laplacian below 10, it was considered “too blurry to analyze. We split this data into development (85% of the population; 67,351 records) and holdout internal validation (15% of the population; 11,885 records) datasets.

#### DB4: Adversarial unlabeled 12-lead ECG images of various formats

To address the challenges of interpreting photographed 12-lead ECG images, influenced by factors such as image quality, capture angle and lighting, we compiled a comprehensive unlabeled dataset of 11,316 12-lead ECG images from different patients. These images were either captured on mobile phones or downloaded from Meta public groups. The images were randomly selected from websites, and none were excluded. After initially splitting the data into development (85% of the population; 9619 records) and holdout (15% of the population; 1697 records) datasets, the development dataset was further divided into training (94% of the development set) and internal validation (6%) datasets.

#### DB5: The China physiological signal challenge 2018 (CPSC2018)

The dataset was introduced as part of a competition to promote the development of algorithms for automated analysis of 12-lead ECG signals, focusing on the accurate detection of arrhythmias. It includes 6879 recordings, each lasting up to 60 s, with a sampling frequency of 500 Hz. Each ECG recording is labeled with one or more diagnostic classes, based on physician interpretations. These annotations cover normal rhythms, various arrhythmias, and other conditions, making the dataset suitable for multi-class classification tasks. The data includes common artifacts, such as baseline wander, powerline interference, and noise caused by muscle movement or electrode misplacement.

### Overview of the deep network model

**Fig. 10:**
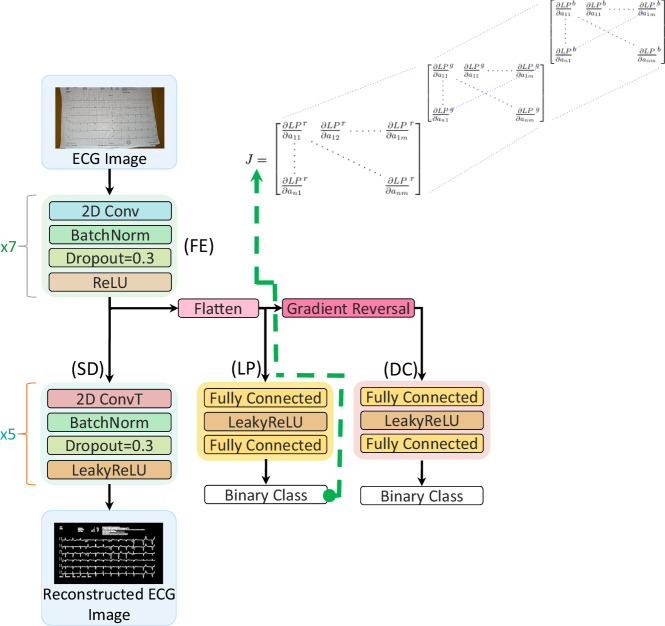
ECG-AIO network architecture. The architecture includes: Feature extractor (FE), Signal Decoder (SD), Label Predictor (LP) and Domain Classifier (DC).

We introduce ECG-AIO, a robust neural network model specifically designed for the classification of photographed 12-lead ECG images. The network employs a transfer learning approach called domain adversarial training and comprises four core components: a joint feature extractor (FE), a signal decoder (SD), a label predictor (LP), and a domain classifier (DC) (see Fig. 10). Not all core components were used for all experiments, as indicated in the description below.

The autoencoder, consisting of the feature extractor and the signal decoder, aims to extract relevant features from ECG images while minimizing information loss. This is especially important in regions with complex patterns, such as shadows. The label predictor and domain classifier utilize the extracted features for their respective tasks. The label predictor extracts features to accurately identify cardiac conditions and provide accurate interpretability knowledge. The purpose of the domain classifier is to determine if the example is an original ECG image or an ECG image photographed using a mobile camera that may include acquisition distortions. Domain classifier training aids in resilience to format changes and to mobile acquisition artifacts.

The feature extractor is a convolutional neural network (CNN) that takes a 2D 12-lead ECG plot as input and extracts relevant features. The configuration of the feature extractor is defined by a set of parameters, including the number of input and hidden channels, kernel sizes, stride, dilation, dropout rate and the application of batch normalization (see Fig. 10).

The signal decoder, a transposed CNN, is designed to extract signals from the 12-lead ECG image. This reconstruction process verifies that the essential details of the original ECG image have been adequately captured by the feature extractor. The parameters that define the decoder are identical to those of the extractor, ensuring symmetry within the autoencoder (see Fig. 10).

After reshaping the feature extractor to a one-dimensional vector, its output was used as the input to both the fully connected label predictor and the fully connected domain classifier. ECG-AIO incorporates a gradient reversal layer between the domain classifier and the feature extractor to enforce domain-agnostic selection of features, thereby achieving independence of ECG image layout and independence of camera artifacts^1^.

The ECG-AIO network was constructed using stacked blocks of convolutional layers, batch normalization, and dropout (see Fig. 10). Both networks employed a leaky linear rectifier (Leaky ReLU) as their activation function. Hyperparameters, including batch size, initial learning rate, number of nodes in the fully connected layers and the number of convolutional layers, were adjusted during training to obtain the optimal model. The initial learning rate was set to 4e-5 for both networks, and the batch size during training was 25.

The internal validation dataset was used to tune the abovementioned hyperparameters.

### Training process

For training, ECG-AIO uses the Adam optimizer^27^, with default parameters β_1_ = 0.9 and β_2_ = 0.999, along with momentum and weight decay regularization. Binary cross-entropy is employed as the loss function for both the label predictor and domain classifier. Meanwhile, mean square error (MSE) loss, also referred to as the L2 distance, is used for the decoder to assess the disparity between the reconstructed and original images. Hyperparameters, such as the learning rate of 4e-5 for the Adam optimizer, were adopted from^1^.

Binary cross-entropy loss is described by:

1 $${\rm{B}}{\rm{C}}{\rm{E}}{\rm{\_}}{\rm{L}}{\rm{o}}{\rm{s}}{\rm{s}}=-\frac{1}{\rm{N}}{\sum_{{\rm{i}}=1}^{\rm{N}}}({\hat{{\rm{y}}}}_{{\rm{i}}}\cdot {\rm{l}}{\rm{o}}{\rm{g}}(\hat{{{\rm{y}}}_{{\rm{i}}}})+(1-{{\rm{y}}}_{{\rm{i}}})\cdot {\rm{l}}{\rm{o}}{\rm{g}}(1-\hat{{{\rm{y}}}_{{\rm{i}}}}))$$

where N is number of samples in the batch, ${y}_{i}$ is the true label for i-th sample (between 0 and 1), and ${\hat{y}}_{i}$ is the predicted probability for the i-th sample, and is a floating-point number between 0 and 1.

Mean square error loss is described by:

2 $$\mathrm{MSE\_Loss}=\frac{1}{H\times W}\sum _{h=1}^{H}\sum _{w=1}^{W}{\left(I\left(h,w\right)-\hat{I}\left(h,w\right)\right)}^{2}$$

where H is the height of the image in pixels, W is width of the image in pixels, I(h,w) is the pixel value at position (h,w) in the original image and $\hat{I}$(h,w) is the pixel value at position (h,w) in the reconstructed image. This formula calculates the average squared difference between corresponding pixels in the grayscale images.

The total loss is described by:

3 $${\mathrm{Loss}}={60}^{* }{\mathrm{Autoencoder}}\_{\mathrm{MSE}}\_{\mathrm{Loss}}+{2}^{* }{\mathrm{Label}}\_{\mathrm{predictor}}\_{\mathrm{BCE}}\_{\mathrm{Loss}}+\,{0.2}^{* }{\mathrm{Domain}}\_{\mathrm{classifier}}\_{\mathrm{BCE}}\_{\mathrm{Loss}}$$

During the training process, the total loss described in Equation (3) is minimized, i.e, all three loss components are reduced simultaneously.

The training process has three main phases aimed at building a robust 12-lead ECG image analysis model:

#### Phase 1 - Signal decoder training

The model starts with random weights, focusing on distinguishing high-quality ECG images from those captured by mobile phone cameras, which often contain artifacts. Through mini-batch training, the signal decoder learns to reconstruct 12-lead ECG signals by isolating the 12-lead ECG signal from background noise using threshold-based segmentation. Mean squared error (MSE) loss measures the difference between predicted and actual 12-lead ECG signals, guiding weight updates. During the first 6 epochs, the signal decoder converges, and a checkpoint of the trained weights is saved.

#### Phase 2 - Condition-specific fine-tuning

This phase introduces a label predictor to detect specific cardiac conditions. Fine-tuning for each cardiac condition was based on supervised learning with labeled 12-lead ECG datasets. A multi-task learning approach is employed, where source images are processed by all model components, while target images pass through only the domain classifier. Losses from the label predictor and domain classifier, along with the gradient reversal layer (GRL), refine the model’s adaptability to source-target domain variations. Separate checkpoints for each cardiac condition are saved.

#### Phase 3 - Domain classifier training

The domain classifier minimizes domain loss (the difference in prediction accuracy between source and target domains) using binary cross-entropy and the GRL, which reverses gradients to ensure the model extracts domain-independent features. This phase aims to generalize the model’s learning for different 12-lead ECG image layouts.

To save the model state (checkpoint) for ECG-AIO, three conditions were required: (1) the label predictor yielded the best heart condition detection result, (2) the signal decoder yielded minimal loss, and (3) the domain classifier accuracy is between 40% and 60%, approximately equal to ‘random guess,’ indicating that the network is agnostic to domain type. To prevent overfitting, network training was halted when the performance on the test set ceased to improve for 5 consecutive epochs. Early stopping conditions were set at 5 epochs during which the training loss decreased but the test loss did not. This threshold was determined empirically. The reason for choosing a trigger larger than 1 is that, in the simplest case, training would stop as soon as the performance on the validation dataset decreases compared to the previous epoch. However, in practice, a larger trigger is needed because neural network training is stochastic and can be noisy. We trained the network with small batches due to GPU memory limitations and the use of high-resolution images. Smaller batches result in a noisier average gradient. Consequently, using relatively small batches introduced some noise into the performance evaluation on the test set. We used two test sets for model training: one that included NYU dataset samples only, used for accuracy assessment of the label predictor and signal decoder of ECG-AIO, and a second one that was an equal mix of NYU dataset images and photographed 12-lead ECG images.

To train the signal encoder, we drew a minibatch from the NYU dataset (DB1), extracted the signal using thresholding (smaller than 10 of 256 (8 bits)), applied augmentation (see below), and forward-propagated it through the network. For the signal encoder, the input is a colored image, and the output is a binary image indicating the presence of an ECG signal. As for the label predictor and domain classifier, the input is a colored image, and the output is a floating-point number indicating a binary outcome. We then back-propagated it through the Label Predictor and updated the Label Predictor and Encoder weights and biases. To train the label predictor, we drew a random mix of samples from the NYU dataset (DB1) and DB3. To train the Domain Classifier, we drew a random mix from all four datasets.

We first trained the signal decoder. After observing convergence over 5 consecutive epochs, we began training the domain classifier concurrently with the signal decoder. At this stage, the model’s objective is to distinguish between photographed and scanned ECG images. The domain classifier is improved by reducing the domain loss, calculated using binary cross-entropy, which measures the disparity between the model’s domain classification and the actual domain label. Upon successful training, a checkpoint is saved, serving as a starting point for subsequent training stages. Importantly, this checkpoint is not condition-specific, allowing it to be used across different condition classification tasks. In the final stage, we simultaneously trained all components of the model—the signal decoder, the label predictor and the domain classifier. The label predictor is introduced into the process, focusing on identifying whether a certain condition is present in the ECG image or not. During this last phase of training, the model adopts a dual-task learning approach. Scanned images are propagated through the signal decoder, label predictor and domain classifier, while mobile-captured images only pass through the domain classifier. By simultaneously training on different types of images and incorporating the domain classifier into the process, the model becomes more adept at handling variations between data sources, leading to enhanced performance and adaptability in real-world scenarios.

To overcome the difference in the convergence rate (i.e., difference in gradient values) between the label predictor and the domain classifier, we used dynamic lambda, as follow:

4 $$\,\lambda =0.85{e}^{5.5p},p=\frac{b+E* {N}_{B}}{{N}_{{Batches}}* {N}_{{Epoch}}},$$

where b is a batch index within an epoch, E is the epoch index, ${N}_{{Batches}}$ is the number of batches in an epoch and ${N}_{{Epoch}}$ is the epoch number. The result is a powerful deep learning model capable of classifying cardiac condition with high accuracy and is agnostic to the data source. This versatility enables the model to effectively handle variations in data origins, rendering it resilient and reliable in real-world scenarios. Note that $\lambda$ is not part of the loss function itself, but is a parameter within the specific model used to evaluate our method. In the model that we used, $\lambda$ is applied to normalize the gradient inversion, as domain adaptation is a comparatively easier task for the network to learn than cardiac disease diagnosis.

Before feature extraction, a segmentation process prepares the scanned ECG images (DB1) by extracting the ECG signal from the image background using a threshold-based method. Following segmentation, shadow augmentations and perspective transformations are applied (see below). The purpose of the training process is to minimize loss between the augmented and segmented images.

To prevent vanishing or exploding gradients, we performed an image normalization process before forward-propagation.

For ECG-AIO, the training time was 60 min per epoch, and a maximum of 50 epochs were used. Early stopping conditions were set at 5 epochs in which the training loss decreased but the test loss did not. The forward-propagation through the DNN took approximately 1 s.

Deep network implementation was performed in Python, using the PyTorch framework (version 2.0.1). In addition, numpy (version 1.24.4) and OpenCV (version 4.8.0.74) libraries were used. The training server consisted of Intel(R) Xeon(R) Gold 6336Y @ 2.4 GHz. RAM: 500 GB. GPU: A40 (of NVIDIA). Cuda version was 12.2. OS: Ubuntu 20.04.6 LTS. Using the abovementioned machine, interpretability calculations take approximately one second. This indicates that the method is feasible for use in a real clinical setting, even with an average (not high-end) machine, where responses can be received within seconds.

### Interpretability mechanism

We introduce a high-resolution interpretability approach designed specifically for 12-lead ECG images, to clarify the decision-making process of our model (Supplementary Fig. 9).

In vector calculus, the Jacobian matrix for a vector-valued function with multiple variables consists of all of its first-order partial derivatives. In this context, it takes the form of a three-dimensional tensor, where each component represents the first-order partial derivative of a specific color channel for a given pixel. Simply put, each element shows how a minor adjustment in the color value of a particular pixel affects the network’s output. For instance, if altering the color of a specific pixel does not impact the output of the network, it indicates that the pixel is not significant for the overall output.

We calculate the Jacobian matrix for the input image based on the label predictor output. The Jacobian matrix captures the first-order partial derivatives of the model concerning each pixel in the image, revealing the sensitivity of the model output to small changes in the input. This indicates how much each pixel contributes to the model’s decision-making process, with the main goal of identifying pixels that have the most significant influence, regardless of whether it is positive or negative. The Jacobian matrix serves as an image, highlighting the relative contribution of each pixel to the outcome.

Our network is binary, classifying each image as either having a specific cardiac condition present or not. This binary nature allows us to quantify how much each pixel in the input image contributes to the final decision. Importantly, we can generate an interpretability image not only for the presence but also for the absence of a cardiac condition. This process provides insights into the specific areas within the ECG image that the model considers important for determining the outcome.

### Prospective clinical experiment

To assess the effectiveness of our algorithm in clinical practice using a perspective experiment, we conducted a comparative analysis between our interpretability images and those manually drawn by two electrophysiologists from different institutes. The study involved images representing one morphological condition (premature ventricular contractions) and one rhythmical condition (atrial flutter). There were 9 images depicting atrial flutter, an additional 9 showing premature ventricular contractions, and one image featuring both conditions.

In the experiment, cardiologists were presented with clinical images and asked to confirm the presence of a specific cardiac condition. After confirming the diagnosis, they were instructed to digitally annotate only the relevant features influencing the diagnosis, while disregarding other possible cardiac conditions. Importantly, the clinical experiment was conducted in a blinded fashion, ensuring that cardiologists remained unaware of the algorithm’s output. A binary matrix summarized the result of this experiment by marking each pixel True if it was marked by the electrophysiologists’ annotation and False otherwise. Identical images (without any superimposed markings) were forward-propagated through the ECG-AIO network and the Jacobian matrix from the ECG-AIO network was computed and scaled to a range of 0–255. A threshold operation was applied to create a second binary matrix, marking all scaled values above 20 as True and all others False. This correlation coefficient calculates the sum of all pixels that are marked by both the clinician and the algorithm, and then divides that sum by the total number of pixels. This approach allows for the measurement of relative similarity between the clinician’s markup and the algorithm’s output, regardless of the image resolution (i.e., the total number of pixels). The correlation coefficient between the two binary matrices was computed using the formula in Equation 2.

5 $$C=\frac{1}{i* j}\left(i* j-\sum _{i=1}^{m}{\sum }_{j=1}^{n}|{a}_{i,j}-{b}_{i,j}|\right)$$

${a}_{i,j}$ represents a pixel in binary matrix of electrophysiologist interpretability, ${b}_{i,j}$ represents a pixel value in a binary matrix derived from Jacobian matrix. Double summation is the L1 distance between two binary images, and denominator of i*j is a normalization factor to scale the coefficient to 0-1 range. When there is no difference between the two images, L1 distance is 0, therefore C = 1. Thus, value of 1 indicates a high correlation between the electrophysiologist’s indications and those of the algorithm, while a value of 0 signifies minimal correlation. The numerator in the formula aggregates all pixels demonstrating correlation—those marked as “True” by both the cardiologist and the algorithm or marked as “False” by both. The denominator enumerates the total number of pixels in the image.

## Supplementary information

Supplement

## Data Availability

The datasets will be freely available on GitHub following publication of the paper (https://technionmail-my.sharepoint.com/:f:/g/personal/yyaniv_technion_ac_il/Eui5xubMPdxJnT1v0ohp4QQBIebH7pb1CW89mH5TX0znbA?e=HKHseq). Please contact Vadim Gliner vadim.gliner@gmail.com.

## Code availability

The data analysis code will be freely available on GitHub following publication of the paper (https://technionmail-my.sharepoint.com/:f:/g/personal/yyaniv_technion_ac_il/Eui5xubMPdxJnT1v0ohp4QQBIebH7pb1CW89mH5TX0znbA?e=HKHseq). Please contact Vadim Gliner vadim.gliner@gmail.com.
