# Supplementary material for: Clinically meaningful interpretability of an AI model for ECG classification
Source: NPJ Digit Med. 2025 Feb 17;8:109. doi: 10.1038/s41746-025-01467-8 (PMC11833077; doi:10.1038/s41746-025-01467-8)
Supplement: Supplementary file 1 — Supplement [file 41746_2025_1467_MOESM1_ESM.docx]

**Clinically meaningful interpretability of an AI model for ECG classification**

**Gliner et al. Clinical interpretability of AI ECG classification**

**SUPPLEMENT**

Vadim Gliner^1^, Idan Levy^1^, Kenta Tsutsui^2^, Moshe Rav Acha^3^, Jorge Schliamser^4^, Assaf Schuster^1^ and Yael Yaniv^5.*^

^1^Computer Science Department, Technion-IIT, Haifa, Israel

^2^Saitama Medical University International Medical Center, Saitama, Japan

^3^Cardiology Department, Shaare Zedek Medical Center, Jerusalem Israel

^4^ Cardiology Department, Lady David Carmel Medical Center, Haifa, Israel

^5^Laboratory of Bioenergetic and Bioelectric Systems, Biomedical Engineering Faculty, Technion-IIT, Haifa, Israel

**^*^Correspondence:**

Yael Yaniv, PhD
Laboratory of Bioenergetic and Bioelectric Systems, Biomedical Engineering Faculty, Technion—IIT, Haifa

Email: [yaely@bm.technion.ac.il](mailto:yaely@bm.technion.ac.il)
Phone: 972-4-8294124
Fax: 972-4-8294599


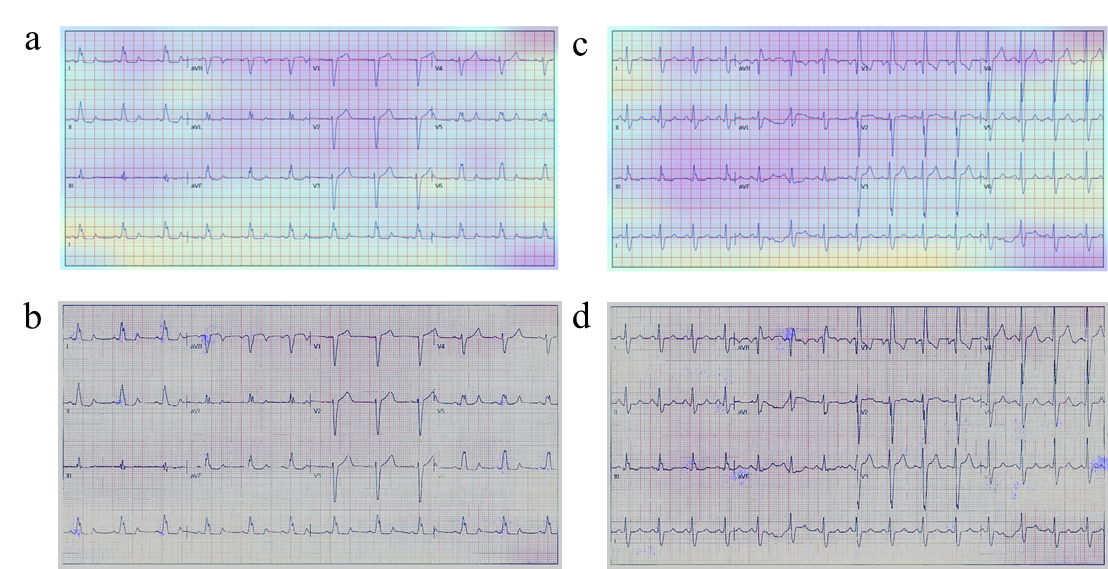


**Supplementary Figure 1:** An example of interpretability on an image with a left bundle branch block identified using (a) a heatmap generated by GRAD-CAM (from^12^) where, evidently, the heatmap focuses on the background and not on the signal and (b) our method which emphasizes the prolonged QRS as well as the dominant S waves. An example of interpretability on an image with a right bundle branch block identified using (c) a heatmap generated by GRAD-CAM (from^12^), where the heatmap again focuses on the background and not on the signal and (d) our method which emphasizes the prolonged QRS, as well as the wide, slurred S waves in the lateral lead (V2) and "M shaped" QRS complexes in the V1 lead.


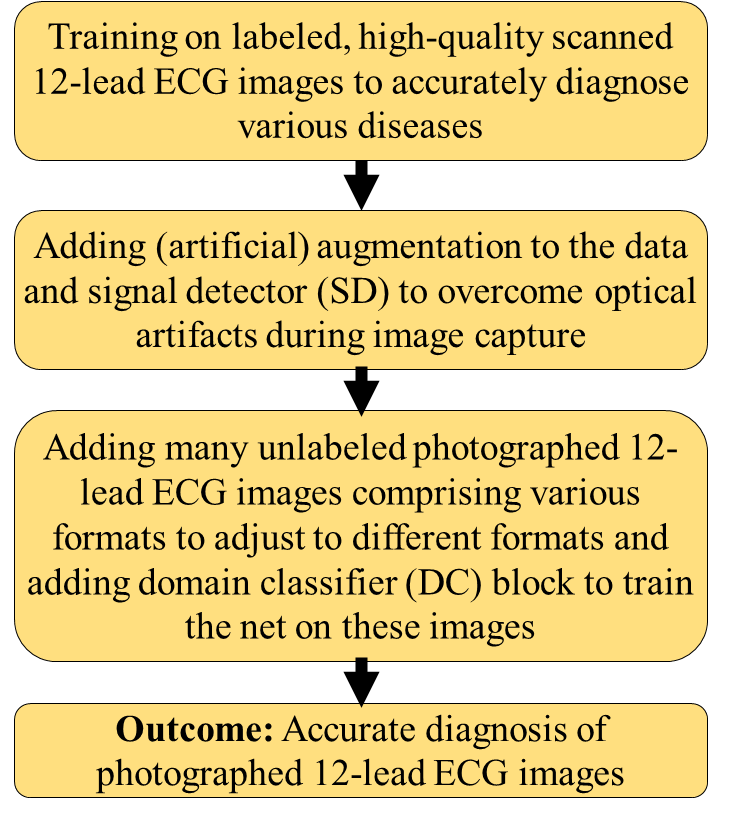


**Supplementary Figure 2:** Schematic diagram of the progression from high-quality scans to photographed images. Schematic diagram from a scan image to an image with interpretability.


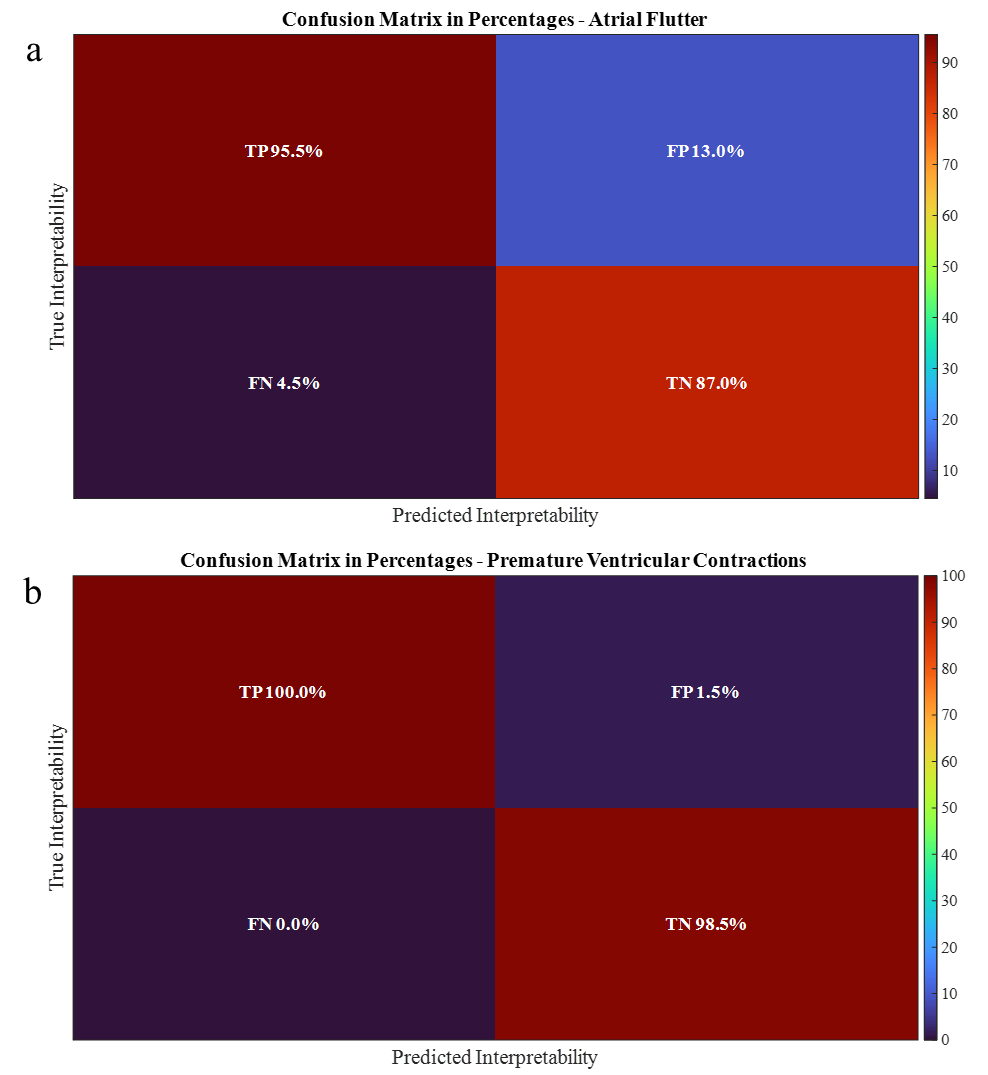


**Supplementary Figure 3:** Confusion matrix of interpretability marker on 200 samples of (a) atrial flutter (rhythmic) and 200 samples of (b) premature ventricular contraction (morphological). A result was defined as a true positive (TP) if 90% or more of the network signal features colocalized with known relevant clinical features of the specific cardiac condition. Conversely, if 90% or more of the network signal features did not localize with relevant clinical features of the specific cardiac condition, it was defined it as a true negative (TN). A result was defined as a false positive (FP) if less than 90% of the network signal features colocalized with relevant clinical features, and as a false negative (FN) if 10% or more of the network signal features localized with irrelevant clinical features of a specific cardiac condition.


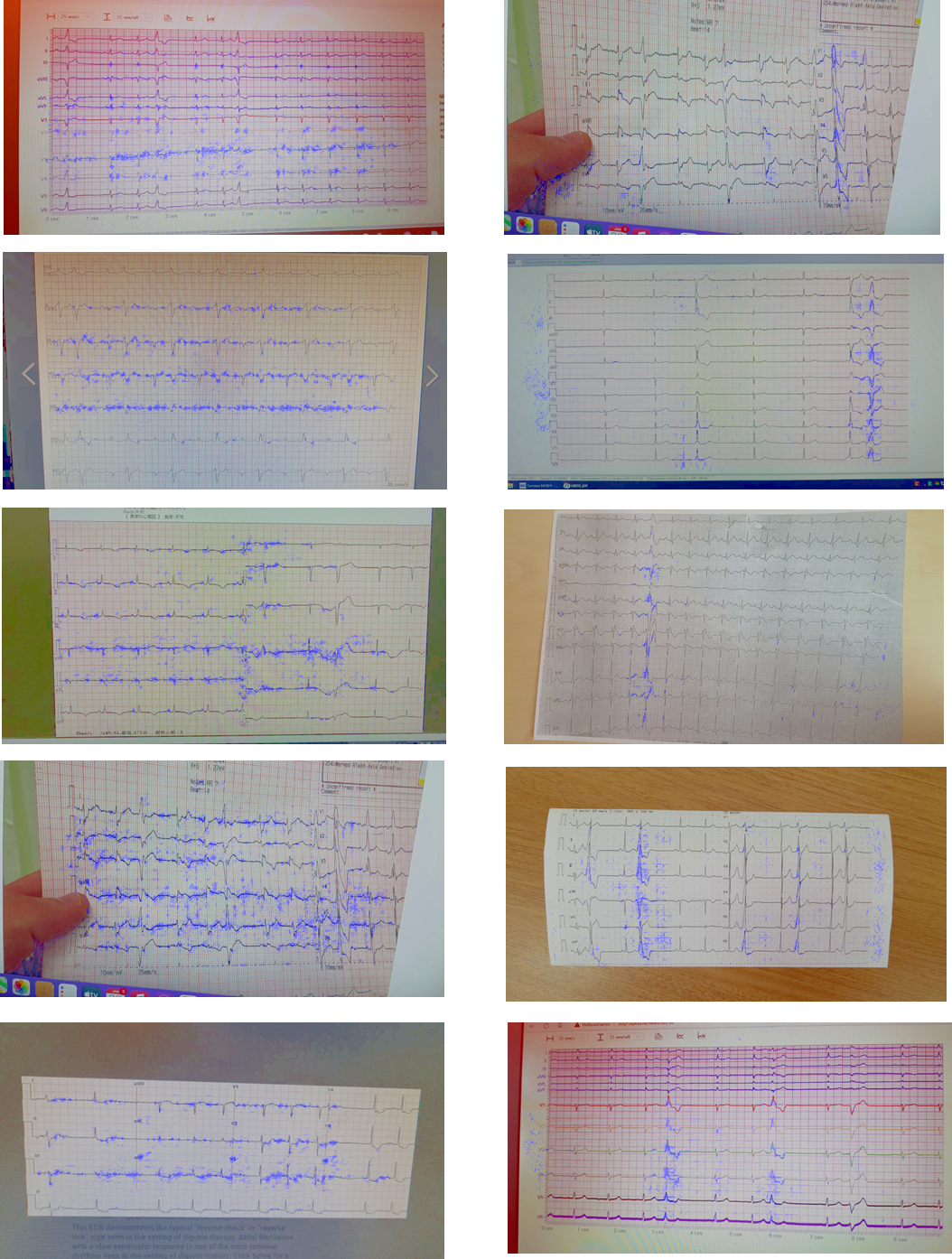


**Supplementary Figure 4:** Interpretability performance of a neural network trained on labeled NYU dataset photographed images (DB3) with shadows and artifacts (DB2) and on unlabeled mobile-captured images from the adversarial database (DB4) and tested on off-axis and rotated photographed images. The interpretability marker is overlaid in purple on the original 12-lead ECG image collected from a patient with atrial flutter (left) and premature ventricular contraction (right).


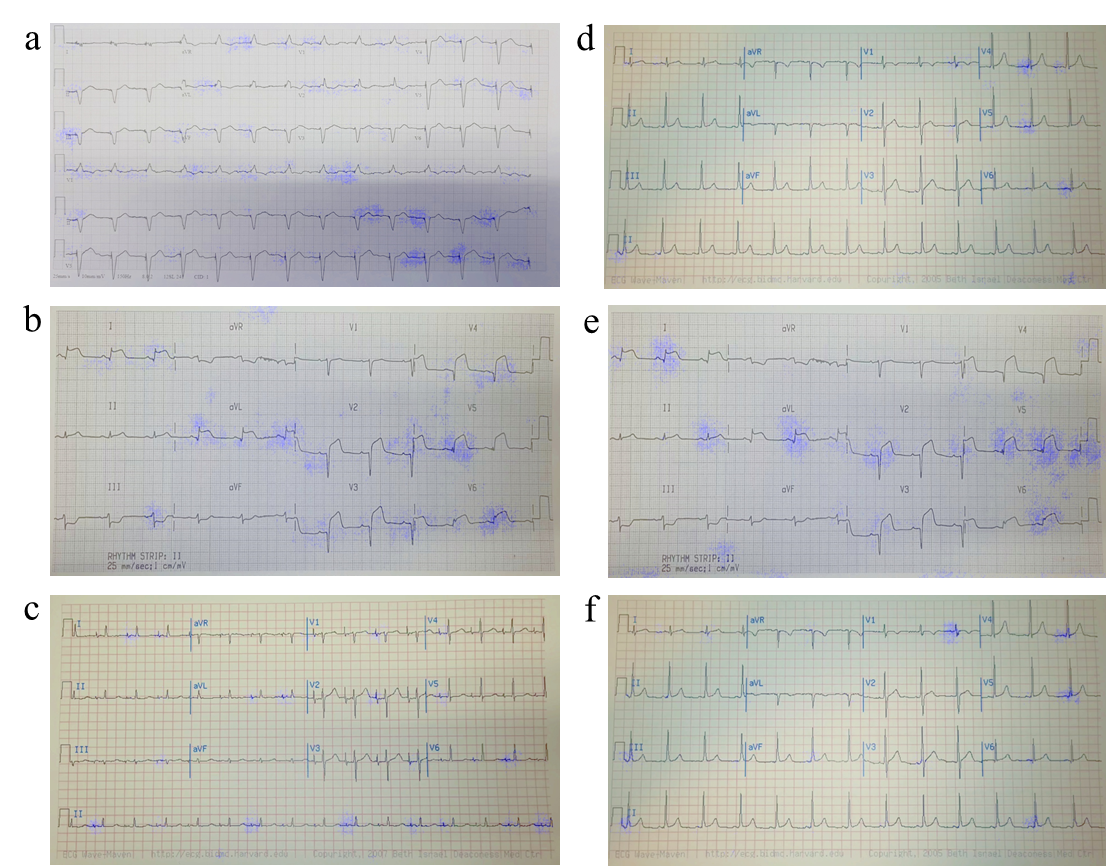


**Supplementary Figure 5:** Interpretability performance of ECG-AIO with a ResNet18-based encoder trained on photographed images (DB3), NYU dataset with shadows and artifacts (DB2), and unlabeled photographed images from the adversarial database (DB4) and tested on photographed images. The interpretability marker is overlaid in purple on the original 12-lead ECG image collected from a patient with (ש) first-degree AV block, (b) myocardial infarction, (c) pacing, (d) PR interval - short, (e) ST elevation, or (f) Wolff-Parkinson-White syndrome. The interpretability mechanism emphasized clinical features on the signal (avoiding the background) that are relevant for each disease.


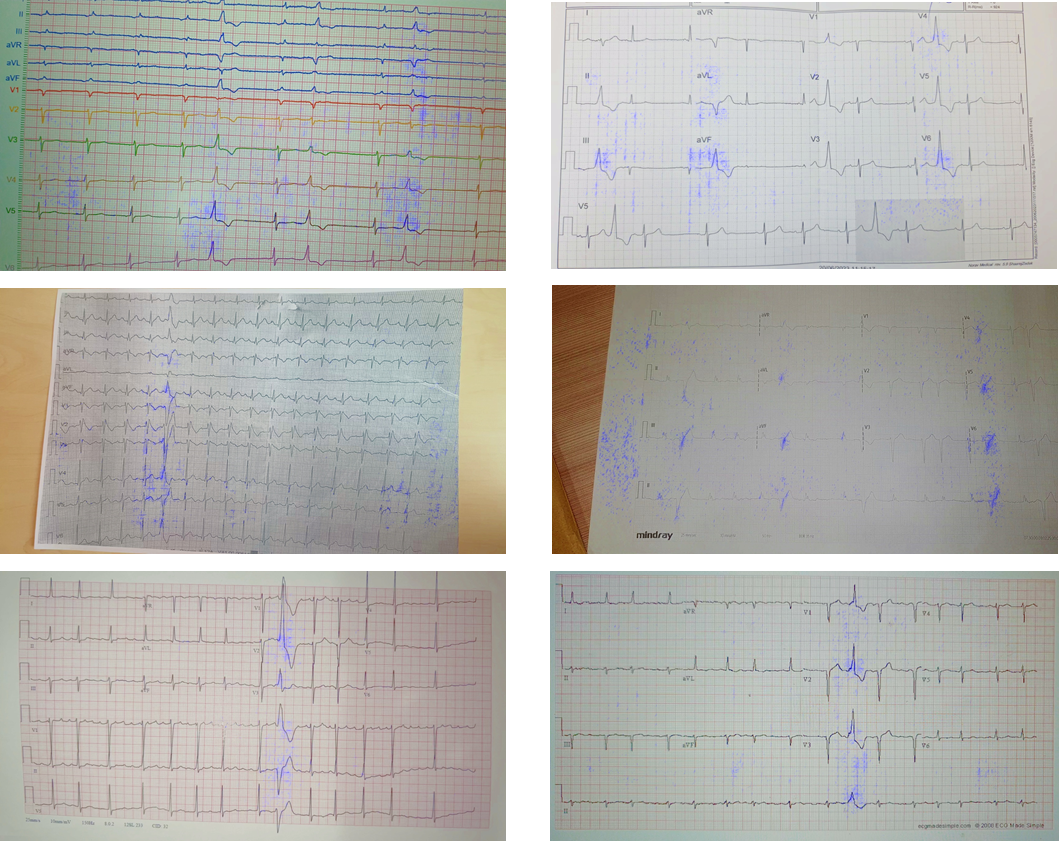


**Supplementary Figure 6:** Interpretability performance of ECG-AIO with a ResNet18-based encoder, tested on photographed images of ECG records of premature ventricular contraction conditions drawn using different layouts.


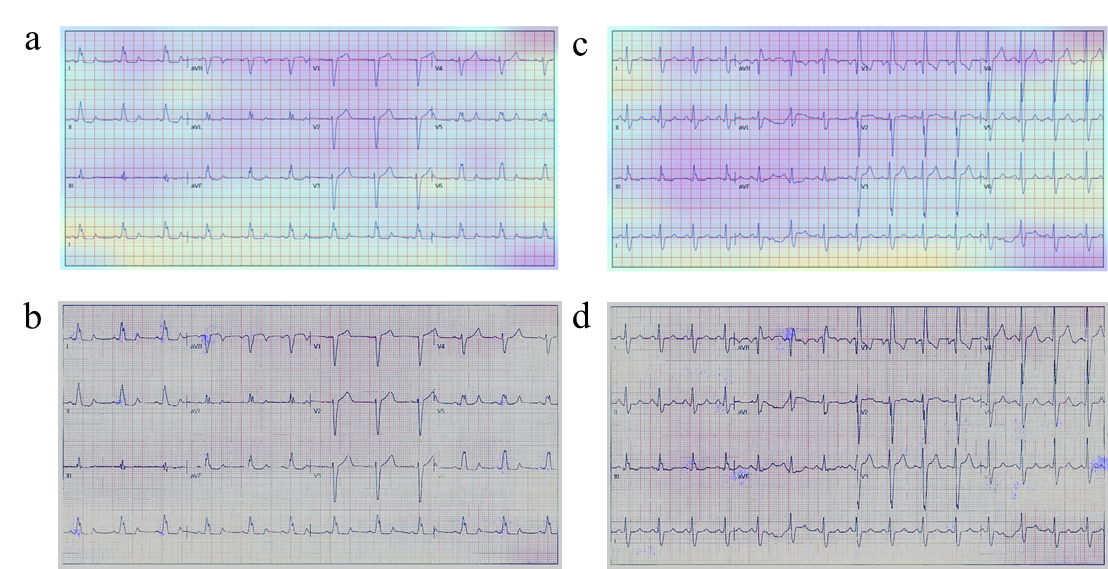


**Supplementary Figure 7:** An example of interpretability on an image with a left bundle branch block identified using (a) a heatmap generated by GRAD-CAM (from^12^), where, evidently, the heatmap focuses on the background and not on the signal and (b) our method which emphasizes the prolonged QRS as well as the dominant S waves. An example of interpretability on an image with a right bundle branch block identified using (c) a heatmap generated by GRAD-CAM (from^12^), where the heatmap focuses on the background and not on the signal and (d) our method which emphasizes the prolonged QRS, as well as the wide, slurred S waves in the lateral lead.


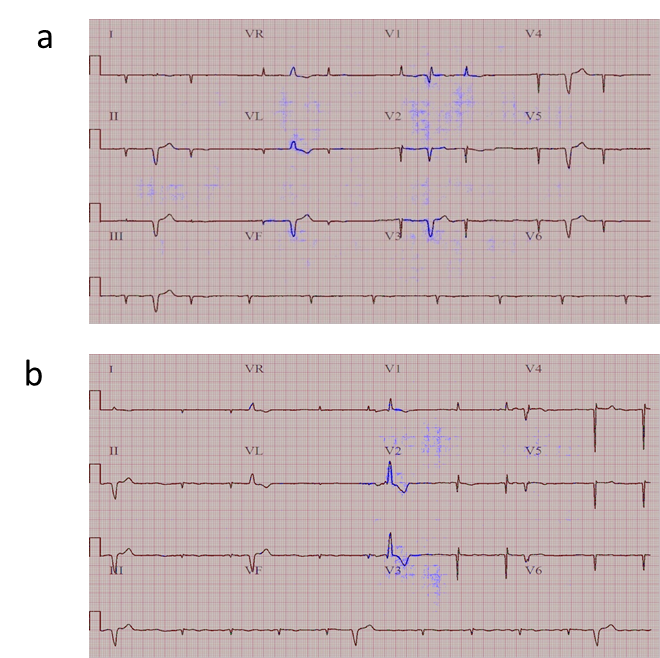


**Supplementary Figure 8:** The first two rendered records from the China 12-lead ECG Challenge database, containing premature ventricular contractions (PVCs), are Record #0025 (A) and Record #0052 (B). In both instances, the interpretability algorithm clearly and effectively highlights the PVC complexes.


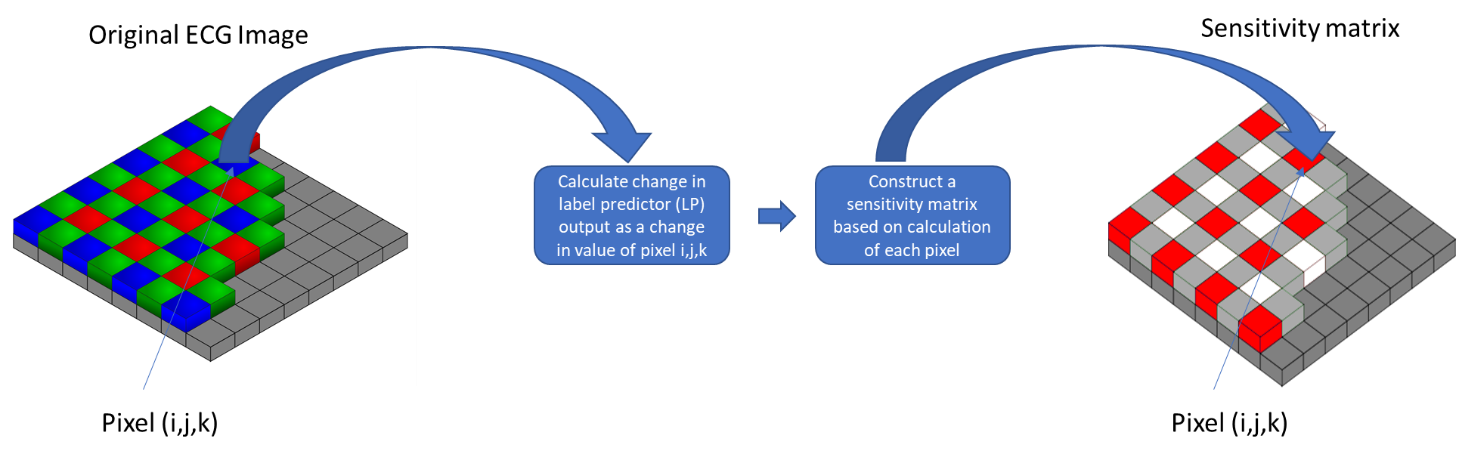


**Supplementary Figure 9:** Schematic description of the interpretability algorithm. From pixel in the original figure to pixel an interpretability figure.

**Supplementary Table 1:** Accuracy of ECG-AIO

| **Category** | **ROC-AUC**  **ECG-AIO** |
| --- | --- |
| Atrial fibrillation | 0.96 |
| Premature ventricular contraction | 0.82 |
| Left axis deviation | 0.88 |
| Left bundle branch block | 0.95 |
| Sinus tachycardia | 0.85 |
| Left atrial enlargement | 0.95 |
| ST changes | 0.88 |
| Left ventricular hypertrophy | 0.93 |
| Sinus bradycardia | 0.95 |
| Sinus arrhythmia | 0.84 |
| ST elevation, myocardial infarction | 0.89 |
| Right bundle branch block | 0.94 |
| Normal variant | 0.95 |
| QT interval, prolonged | 0.96 |
| **Average** | **0.91** |

**Supplementary Table 2:** The number of positive and negative samples for each category in DB1.

| Category | NY_True | NY_False |
| --- | --- | --- |
| Atrial Fibrillation | 3055 | 76171 |
| Atrial Flutter | 603 | 78623 |
| AV Block - First-degree | 515 | 78711 |
| Left Axis Deviation | 4561 | 74665 |
| Left Bundle Branch Block | 2853 | 76373 |
| Left Ventricular Hypertrophy | 8070 | 71156 |
| Premature Ventricular Contractions | 3213 | 76013 |
| Pacing | 1586 | 77640 |
| Sinus Bradycardia | 7213 | 72013 |
| Sinus Tachycardia | 10189 | 69037 |
| Premature Atrial Contractions | 2351 | 76875 |
| Wolff-Parkinson-White | 43 | 79183 |
| Right Bundle Branch Block - General | 6494 | 72732 |
